# Enhancement of Phytochemicals and Antioxidant Activity of Thai Fermented Soybean Using Box–Behnken Design Guided Microwave-Assisted Extraction

**DOI:** 10.3390/foods14152603

**Published:** 2025-07-24

**Authors:** Piya Temviriyanukul, Woorawee Inthachat, Ararat Jaiaree, Jirarat Karinchai, Pensiri Buacheen, Supachai Yodkeeree, Tanongsak Laowanitwattana, Teera Chewonarin, Uthaiwan Suttisansanee, Arisa Imsumran, Ariyaphong Wongnoppavich, Pornsiri Pitchakarn

**Affiliations:** 1 Institute of Nutrition, Mahidol University, Salaya 73170, Nakhon Pathom, Thailand; piya.tem@mahidol.ac.th (P.T.); woorawee.int@mahidol.ac.th (W.I.); uthaiwan.sut@mahidol.ac.th (U.S.); 2 Department of Biochemistry, Faculty of Medicine, Chiang Mai University, Muang Chiang Mai 50200, Chiang Mai, Thailand; ararat.ja@gmail.com (A.J.); jirarat.karin@gmail.com (J.K.); pensiri.bua@cmu.ac.th (P.B.); supachai.y@cmu.ac.th (S.Y.); tanongsak.l@cmu.ac.th (T.L.); teera.c@cmu.ac.th (T.C.); arisa.bonness@cmu.ac.th (A.I.); ariyaphong.w@cmu.ac.th (A.W.)

**Keywords:** Thua-Nao, insulin resistance, diabetes, α-glucosidase, α-amylase, isoflavones

## Abstract

Thai fermented soybeans (TFSs) contain phytochemicals with anti-diabetic benefits. In this study, an initial non-optimized TFS extract (TFSE) was prepared using a conventional triplicate 80% ethanol extraction method and evaluated for its biological activity. TFSE effectively reversed TNF-α-induced insulin resistance in 3T3-L1 adipocytes by enhancing insulin-stimulated glucose uptake, indicating anti-diabetic potential. TFSE also upregulated the phosphorylation of AKT (a key insulin signaling mediator) and the expression of adipogenic proteins (PPARγ, CEBPα) in TNF-α-exposed 3T3-L1, suggesting the mitigation of adipocyte dysfunction; however, the results did not reach statistical significance. The conventional extraction process was labor-intensive and time-consuming, and to enhance extraction efficiency and bioactivity, the process was subsequently optimized using environmentally friendly microwave-assisted extraction (MAE) in combination with the Box–Behnken design (BBD) and response surface methodology (RSM). The optimized extract (O-TFSE) was obtained over a significantly shorter extraction time and exhibited higher levels of total flavonoids and antioxidant activity in comparison to TFSE, while showing reduced levels of isoflavones (daidzein, genistein, and glycitein) in relation to TFSE. Interestingly, O-TFSE retained similar efficacy in reversing TNF-α-induced insulin resistance and demonstrated significantly stronger α-glucosidase and α-amylase inhibitory activities, indicating its enhanced potential for diabetes management. These results support the use of MAE as an efficient method for extracting functional compounds from TFS for functional foods targeting insulin resistance and type 2 diabetes mellitus.

## 1. Introduction

Projected figures from the International Diabetes Federation (IDF) indicate that 783 million adults will develop type 2 diabetes mellitus (T2DM) by 2045 if the current trends continue [1]. T2DM is a widespread chronic disease driven by factors like increasing obesity, physical inactivity, and an aging population [2,3,4]. The condition arises from the inability of peripheral tissues to respond to insulin and insufficient insulin production by pancreatic β-cells, leading to high blood sugar and serious complications such as heart disease, neuropathy, retinopathy, and kidney disease [5,6,7,8,9]. The expenses associated with diabetes management constitute a considerable financial and social burden. Although types of medication, including metformin, sulfonylureas, thiazolidinediones, and α-glucosidase inhibitors (AGIs), are available, many are often associated with undesirable side effects, such as gastrointestinal problems, hypoglycemia, and increased cardiovascular risks [10,11,12,13]. As a result, many T2DM patients ultimately require complex treatment regimens involving drug combination or insulin injections, often lowering quality of life. These challenges have encouraged the search for alternative or supplementary approaches, particularly those originating from natural products and functional dietary regimens [14,15,16].

Oxidative stress is a key contributor to the onset and progression of insulin resistance and T2DM [17]. Reactive oxygen species (ROS) are usually generated as byproducts of normal cellular metabolism. Elevated ROS levels in obesity and hyperglycemia result from mitochondrial dysfunction and the activation of pro-inflammatory pathways [18,19], while the excessive production of ROS can damage cellular macromolecules, impair insulin signaling, and promote dysfunction in pancreatic β-cells [20,21]. Antioxidants, which inhibit the excessive formation of ROS, have therefore emerged as promising agents for preventing or delaying diabetes-related complications [22,23]. Epidemiological studies have also demonstrated that diets rich in fruits, vegetables, and whole grains, which are natural sources of antioxidants, help lower the risk of T2DM [24,25]. Among dietary antioxidants, polyphenols, such as flavonoids and isoflavones, have been particularly well studied [26,27]. Legumes like soybeans are abundant in these compounds, which are known to scavenge free radicals, prevent inflammation, and enhance glucose utilization [28,29]. The bioavailability and bioactivity of these phytochemicals are influenced by their chemical form, the food matrix, and the processing techniques used [30], highlighting the significance of optimizing extraction and preparation techniques.

Fermented foods are recognized not only for their food preservation qualities but also their health benefits due to bioactive peptides, organic acids, and enhanced levels of vitamins and phytochemicals [31]. Popular fermented soybean products in Asia such as Natto, tempeh, and Thua-Nao, have been associated with lower risks of chronic illnesses, such as diabetes and heart disease [32]. Thua-Nao, a traditional fermented soybean product from Northern Thailand, is produced by fermenting cooked soybeans with naturally occurring *Bacillus subtilis* and other microorganisms [33]. The fermentation process enhances the digestibility and flavor of soybeans and greatly increases the concentration of bioactive isoflavones, particularly the aglycone forms including daidzein, genistein, and glycitein [33]. These forms are more easily absorbed and biologically active than their glycoside precursors [34]. Recent in vitro and in vivo studies have indicated that Thua-Nao extracts can suppress inflammation and carcinogenesis and protect against cell impairment [35,36], supporting its positive biological effects on several chronic conditions including insulin resistance and diabetes.

Extracting bioactive compounds from plant materials is a critical step in the development of functional foods and nutraceuticals. Traditional methods such as maceration, Soxhlet extraction, and percolation are often time-consuming, require large amounts of organic solvents, and can degrade heat-sensitive compounds [37]. Due to these drawbacks, alternative extraction technologies have been developed, including ultrasound-assisted extraction (UAE), supercritical fluid extraction (SFE), and microwave-assisted extraction (MAE) [38]. MAE is increasingly popular as it can rapidly and evenly heat samples, thereby improving extraction efficiency, reducing solvent use, and shortening processing times [39,40]. The mechanism of MAE involves the interaction of microwave energy with polar molecules in the solvent and sample, resulting in localized heating and better mass transfer [41]. The effectiveness of MAE depends on several factors such as the solvent type and concentration, microwave power, extraction duration, and the ratio of sample to solvent [42]. The optimization of these factors is essential for maximizing the yield and bioactivity of target compounds while minimizing energy and solvent requirements.

Optimizing extraction is complicated due to the need to evaluate multiple variables and their interactions. Traditional one-factor-at-a-time (OFAT) approaches are inefficient because they overlook how variables interact [43]. Response surface methodology (RSM) is a powerful statistical tool that allows for the simultaneous evaluation of multiple variables and their impact on response variables such as extraction yield, antioxidant activity, and phytochemical content [44]. The Box–Behnken design (BBD), a widely used RSM approach, requires fewer experimental runs than full factorial designs while still providing reliable models for optimization [45]. BBD has been successfully applied to optimize the extraction of bioactive compounds from various plant materials, including *Diplazium esculentum* (Retz.) Sw., *Citrus maxima* Albedo, *Aeginetia indica* L., *Moringa oleifera* leaves, citrus peels, and medicinal herbs [46,47,48].

This study is structured in two phases: first, an investigation into the biological activity of a non-optimized Thai fermented soybean extract (TFSE) produced by conventional ethanol extraction, and second, optimization of the extraction process using MAE in combination with BBD and RSM to enhance yield and bioactivity. The optimized extract (O-TFSE) was then characterized for its phytochemical profile and evaluated for antioxidant and anti-diabetic activities, specifically its inhibition of α-glucosidase and α-amylase, and its ability to reverse insulin resistance in TNF-α-treated 3T3-L1 adipocytes. This two-phase strategy offers a methodological framework for efficiently extracting functional ingredients from traditional fermented foods. The results are significant for the development of innovative functional foods and nutraceuticals aimed at preventing oxidative stress-related diseases, insulin resistance, and T2DM.

## 2. Materials and Methods

### 2.1. Chemicals and Reagents

Calf serum (CS), Dulbecco’s modified Eagle’s medium (DMEM), penicillin/streptomycin, and trypsin EDTA were purchased from Gibco (Grand Island, NY, USA). Fetal bovine serum (FBS) was supplied by Hyclone (Logan, UT, USA). Anti-phospho-Akt (Ser473), anti-AKT, anti-C/EBPα, and anti-PPARγ (81B8) antibodies were obtained from Cell Signaling Technology (Danvers, MA, USA). The antibody specific to β-actin was purchased from Sigma-Aldrich (St. Louis, MO, USA). HPLC standards, including daidzin, daidzein, genistein, genistin, glycitein, and glycitin (all with HPLC purity >98%) were obtained from Biopurify Phytochemicals (Chengdu, China). HPLC standards for caffeic acid, catechin hydrate, ferulic acid, gallic acid, 4-hydroxybenzoic acid, and protocatechuic acid (all with HPLC purity >98%) were acquired from Sigma-Aldrich (St. Louis, MO, USA). Standards for LC–ESI–MS/MS including daidzein (≥98.0% HPLC), daidzin (≥98.0% HPLC), glycitein (≥98.0% HPLC), glycitin (≥98.0% HPLC), and genistin (≥98.0% HPLC), were obtained from Tokyo Chemical Industry (Tokyo, Japan). The Amersham™ Protran Premium 0.2 µm NC nitrocellulose blotting membrane was supplied by Cytiva (Cytiva, Dassel, Germany), and ECL reagents were supplied by Bio-Rad (Bio-Rad Laboratories, Watford, UK).

### 2.2. Plant Sample and Thai Fermented Soybean (TFS) Preparation

Soybeans were obtained from a commercial brand in Thailand. The Thai fermented soybean, known as Thua Nao or TFS, was prepared using a traditional method. Briefly, the soybeans were soaked in water for 12 h, then boiled for 3 h, and finally cooled to 40 °C. The cooked soybeans underwent a natural fermentation process that lasted three days at a temperature of 37 °C and a humidity level of 80%.

### 2.3. Crude TFS Extract Preparation by Conventional Extraction

The TFS was dehydrated at 50 °C using a hot-air oven and then ground into a powder. The powder was subjected to extraction three times with 80% (*v*/*v*) ethanol; soaking took place overnight at room temperature. The supernatant was collected, then concentrated, and freeze-dried using vacuum evaporation and lyophilization, respectively, to obtain the crude ethanolic extract of TFS (TFSE) as a powder, which represents a non-optimized extract. The ethanolic extract of TFS was kept at −20 °C until further use.

### 2.4. Quantification of Phenolic and Isoflavone Derivative Contents Using HPLC Analysis

The measurement of phenolics, including caffeic acid, catechin hydrate, ferulic acid, gallic acid, 4-hydroxybenzoic acid, protocatechuic acid, and isoflavone derivatives daidzein, daidzin, genistein, genistin, glycitein, and glycitin, was carried out using high-performance liquid chromatography (HPLC). For analysis, the extract was prepared at a 10 mg/mL concentration in HPLC-grade methanol, mixed by vortexing for 1 min before being sonicated for 10 min and centrifuged at 10,000 rpm for 5 min. The resulting supernatant was then passed through a 0.22 μm nylon membrane filter. HPLC was performed on an Agilent 1260 Infinity II system (Agilent Technologies, Santa Clara, CA, USA) equipped with a ZORBAX Eclipse Plus C18 column (95 Å, 5 μm, 4.6 × 250 mm; Agilent Technologies), with the column maintained at 25 °C. The mobile phase used solvent A (0.1% formic acid in deionized water) and solvent B (acetonitrile), following a programmed gradient elution: 0–2.5 min, 100–88% A; 2.5–5 min, 88% A; 5–7 min, 88–82% A; 7–10 min, 82% A; 10–13 min, 82–74% A; 13–19 min, 74% A; 19–23 min, 74–54% A; 23–26 min, 54% A; 26–30 min, 54–25% A; 30–32 min, 25–0% A; and 32–35 min, 0% A, with a corresponding increase in solvent B. The flow rate was set at 0.8 mL/min, and each sample’s injection volume was 10 μL. Detection was accomplished at 254 nm using a diode array detector. The compounds were quantified by comparing the peak areas to those of external standards calibration curves. Data were collected and analyzed with OpenLAB CDS ChemStation Edition Software version 3.5 (3.5.0). To confirm the specificity of the measurement and to exclude interference from co-eluting compounds, each analyte’s retention time and UV spectrum were matched to authentic standards. The chromatographic procedure was optimized to ensure the clear separation of each compound. Where applicable, spiking and recovery tests were conducted to validate the absence of matrix interference.

### 2.5. Cells and Cell Culture

3T3-L1 cells were obtained from the Japanese Collection of Research Bioresources (JCRB). For cell maintenance and proliferation, they were grown as preadipocytes in DMEM supplemented with L-glutamine, 10% calf serum (CS), and 1% penicillin, maintained under 5% CO_2_ at 37 °C. To induce differentiation into mature adipocytes, confluent 3T3-L1 cells were treated with an induction medium containing 0.5 mM 3-isobutyl-1-methylxanthine (IBMX), 1 µM dexamethasone, 1 µg/mL insulin, and 10% fetal bovine serum (FBS) for three days. Then, the cells were further exposed to a differentiation medium with 1 µg/mL insulin and 10% FBS for another three days. Finally, the adipocyte maturation was completed by incubating the cells in a maturation medium containing 0.5 µg/mL insulin and 10% FBS for an additional six days.

### 2.6. Cytotoxicity Testing

3T3-L1 preadipocytes were seeded into 96-well plates at a density of 3000 cells/well and incubated for 48 h. Following this, the cells underwent differentiation and maturation using the induction, differentiation, and maturation media as described in Section 2.3. The mature adipocytes were treated with various concentrations (0–400 µg/mL) of either the non-optimized crude ethanolic fraction (TFSE) or optimized TFSE (O-TSFE) for 72 h. Cell viability was assessed using the sulforhodamine B (SRB) assay. After treatment, cells were fixed with 10% (*w*/*v*) trichloroacetic acid and stained with SRB for 30 min. Excess dye was removed by repeatedly washing the cells with 1% (*v*/*v*) acetic acid. The protein-bound dye was then dissolved using a 10 mM Tris base solution, and the absorbance was measured at 510 nm with a Synergy Hybrid microplate reader (BioTek Instruments, Inc., Winooski, VT, USA).

The percentage cell survival was calculated as follows.

(1) $$\%\;Cellular\;Viability=\frac{(Abs\;of\;treatment\;group)\times 100}{\left(Abs\;of\;control\;group\right)}$$

An inhibitory concentration of 20% (IC20) or a non-toxic concentration was used for further experiments.

### 2.7. Determination of Anti-Insulin Resistance Activity Using Cellular Glucose Uptake Assay

In this experiment, TNF-α was used as an inflammatory cytokine to induce insulin resistance, resulting in reduced glucose uptake in adipocytes. Mature 3T3-L1 adipocytes were treated with 2 ng/mL TNF-α along with either TFSE or O-TSFE (0–400 µg/mL) for 72 h. After treatment, glucose uptake was assessed using the fluorescent glucose analog 2-(N-(7-Nitrobenz-2-oxa-1,3-diazol-4-yl)Amino)-2-Deoxyglucose (2-NBDG). Cells were incubated in serum-free, low-glucose DMEM for 3 h at 37 °C, then washed with PBS and centrifuged at 6000 rpm at 4 °C. Subsequently, they were incubated with 80 µM 2-NBDG, with or without 50 nM insulin, for 1 h at 37 °C. Following washing, intracellular fluorescence was captured using an inverted fluorescence microscope (ZEISS Axio Observer 7, Carl Zeiss Microscopy, Jena, Germany) to measure the uptake of the fluorescent glucose analog. Ten micromolar of pioglitazone (POZ) served as a positive control for insulin-sensitizing activity (Figure S2), while DMSO acted as a vehicle (negative) control.

### 2.8. Measurement of Phosphorylation of AKT and Expression of C/EBPα and PPARγ via Western Blotting

Following the treatment described in Section 2.8, cells were collected to analyze the expression of differentiation signaling proteins. Protein lysates were prepared and separated using 10% SDS-PAGE, after which the proteins were transferred onto nitrocellulose membranes. The membranes were then probed with specific antibodies: the anti-phospho-Akt (Ser473) antibody at a dilution of 1:1000 (Cell Signaling, USA), anti-AKT antibody at a dilution of 1:2000 (Cell Signaling, USA), anti-C/EBPα at a dilution of 1:1000 (Cell Signaling, USA), anti-PPARγ (81B8) at a dilution of 1:1000 (Cell Signaling, USA), and anti-β-actin antibody at a dilution of 1:10,000 (Sigma-Aldrich, USA). The bands were detected at molecular weights of 60 kDa for phospho-Akt (Ser473) and AKT, 42 kDa for C/EBPα and β-actin, and 53–57 kDa for PPARγ.

### 2.9. Box–Behnken Design (BBD) and Response Surface Methodology (RSM) for the Optimization of Microwave-Assisted Extraction

In this study, three extraction factors were investigated for microwave extraction: ethanol concentration (X1, % *v*/*v*), microwave power (X_2_, Watt (W)), and extraction time (X_3_, second (sec)). The actual levels for ethanol concentration (40, 60, and 80% *v*/*v*), microwave power (100, 200, and 500 W), and extraction time (30, 60, and 90 s) were selected based on preliminary experiments and previous studies to cover a suitable range for each factor [38,39,40,41,42,43,44,45,46,47,48], as detailed in Table 1. A total of 15 experimental runs derived from the BBD are shown in Table 2. The optimization utilized several response variables: total phenolic content (TPC), total flavonoid content (TFC), isoflavones (daidzein, genistein, and glycitein), and antioxidant activities were measured using ORAC, FRAP, and DPPH assays. These variables were selected to ensure that the extraction process would maximize the yields of bioactive compounds and antioxidant properties.

### 2.10. Determination of Total Phenolic Content (TPC), Total Flavonoid Content (TFC), and Antioxidant Activities

TPC was measured using the Folin–Ciocalteu assay, following an established protocol from a previous study [49], with gallic acid as the reference standard, and absorbance measured at 765 nm. Results were expressed as milligrams of gallic acid equivalents per gram of dry weight (mg of GAE/g DW).

TFC was assessed using sodium nitrite and aluminum chloride hexahydrate based on a standard protocol [50]. Absorbance was read at 510 nm, and TFC was quantified as milligrams of quercetin equivalents per gram of dry weight (mg QE/g DW), using a calibration curve ranging from 10 to 1000 mg/mL of quercetin.

Antioxidant activity was evaluated using three common assays: 2,2-diphenyl-1-picrylhydrazyl (DPPH) radical scavenging, ferric reducing antioxidant power (FRAP), and oxygen radical absorbance capacity (ORAC), as described in the method used by Luu et al. (2023) [51], without modifications. Trolox was used as the standard, and antioxidant capacity was reported in micromoles of Trolox equivalents per gram of dry weight (µmol TEAC/g DW). All measurements were performed with a Synergy™ HT 96-well UV-visible microplate reader and Gen 5 data analysis software (BioTek Instruments, Inc., Winooski, VT, USA).

### 2.11. Determination of Enzyme Inhibitory Activity

#### 2.11.1. α-Glucosidase Inhibition Assay

An α-glucosidase inhibition assay was conducted using a 96-well microplate. Briefly, 10 µL of α-glucosidase solution (0.2 U/mL) from *Saccharomyces cerevisiae* was pre-incubated with 5 µL of the sample extract and 160 µL of potassium phosphate buffer (KPB, pH 7.0) at 37 °C. The reaction was initiated by adding 25 µL of 10 mM p-nitrophenyl-α-D-glucopyranoside (pNPG) as the substrate. After the incubation period, the formation of p-nitrophenol was quantified by measuring absorbance at 405 nm using a Synergy™ HT microplate reader (BioTek Instruments, Inc., Winooski, VT, USA) [51]. Enzyme inhibition was calculated as the percentage reduction in α-glucosidase activity compared to the control wells (enzyme without extract). Miglitol and acarbose served as positive controls.

#### 2.11.2. α-Amylase Inhibition Assay

The inhibitory effect on α-amylase was assessed using porcine pancreatic α-amylase (type VII, ≥10 U/mg). For the assay, 100 µL of α-amylase solution (50 mg/mL) was combined with 50 µL of the sample extract in each well of a 96-well plate. The enzymatic reaction was initiated by adding 50 µL of 30 mM p-nitrophenyl-α-D-maltohexaoside (pNPM) as the substrate. The hydrolysis of pNPM was monitored by measuring absorbance at 405 nm. The percentage of enzyme inhibition was determined by comparing the reaction rates between wells containing sample and control wells (enzyme without extract) [51]. Acarbose was used as the positive control.

### 2.12. Metabolomic Profiling of Phytochemicals of TFSE and O-TFSE Using HPLC-qTOF-MS

Non-targeted metabolomic profiling was conducted as described in previous studies [46,47]. In brief, TSFE or O-TFSE samples were dissolved in 0.1% (*v*/*v*) DMSO and injected into an Acclaim™ RSLC 120 C18 column (100 mm × 2.1 mm, 2.2 µm particle size, 120 Å pore size; Thermo Fisher Scientific, Waltham, MA, USA), which was connected to an HPLC-qTOF-MS system (TripleTOF^®^ 6600 quadrupole time-of-flight mass spectrometer; AB Sciex, Framingham, MA, USA). The mobile phase used solvent A (0.1% formic acid in water) and solvent B (0.1% formic acid in acetonitrile). Chromatographic separation was carried out at a flow rate of 0.4 mL/min over 30 min. Mass spectra were acquired in both positive and negative ion full scan modes, covering a mass range of 100–1000 *m*/*z*. The collected data were processed with the SCIEX OS software, version 3.3.0 platform (AB Sciex) and compared with reference spectra from the High-Resolution MS/MS Spectral Library and the National Institute of Standards and Technology (NIST) database.

### 2.13. Molecular Docking and Molecular Dynamics (MD) Simulation

The crystallographic structure of yeast α-glucosidase (PDB: 3A4A), obtained from the NCBI database, was employed as the template for docking studies. Isoflavone and diabetes drug structures were retrieved from PubChem. Docking these compounds with the enzyme active site was performed using UCSF Chimera (version 1.19) with the box size set at 40 × 40 × 40 and the grid box size set at 22 × −5 × 23 (angstrom, Å). To validate the binding interactions, molecular dynamics simulations were conducted using GROMACS version 2018. The protein–ligand complexes were parameterized with the CHARMM36 force field, solvated in a cubic box with TIP3P water molecules, and neutralized with counter ions. Structural stability and dynamics were assessed over 500 picoseconds by calculating the root mean square deviation (RMSD), root mean square fluctuation (RMSF), and radius of gyration (Rg).

### 2.14. Statistical Analysis

Statistical analysis was performed using Design-Expert version 13 (Stat-Ease Inc., Minneapolis, MN, USA) for BBD and RSM through experimental design, regression, and graphical analysis. All experiments were conducted in triplicate and reported as the mean ± standard deviation (SD). Differences between individual groups were analyzed using either Student’s *t*-test or one-way analysis of variance (ANOVA), followed by Tukey’s multiple comparisons test. (GraphPad Prism version 10.4.2, GraphPad Software, Boston, MA, USA). *p* Values < 0.05 were regarded as statistically significant.

## 3. Results

### 3.1. The Ethanolic Extract of Thai Fermented Soybean (TFSE) Suppresses Inflammation-Induced Insulin Resistance in TNF-α-Treated 3T3-L1 Adipocytes

**Figure 1:**
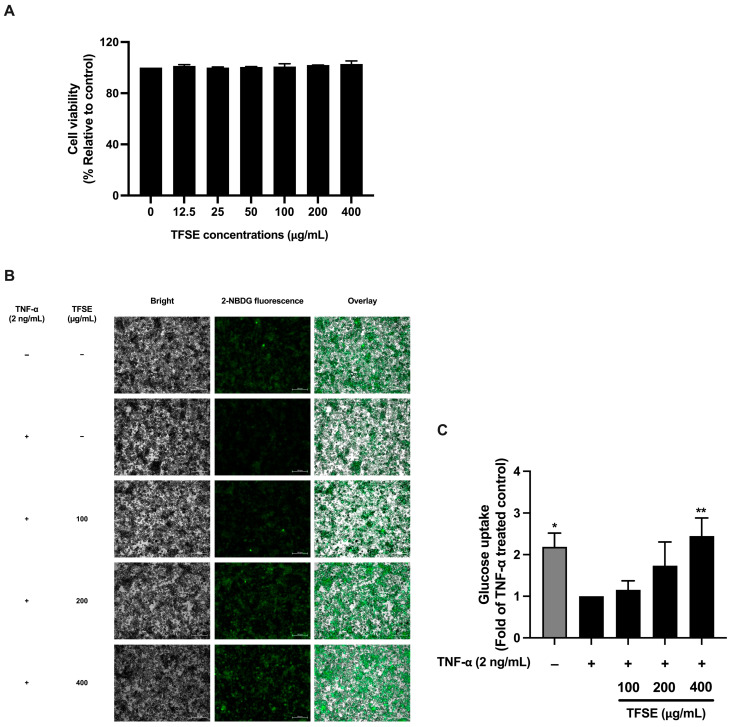
TFSE mitigates inflammation-induced insulin resistance. The cytotoxicity of TFSE in mature 3T3-L1 adipocytes determined by the SRB assay (**A**). The cells were treated with various doses of TFSE (12.5–400 µg/mL) for 72 h. The effect of TFSE on insulin-induced cellular glucose uptake in TNF-α-treated 3T3-L1 adipocytes (**B**,**C**). Fluorescent microscopic images of cells (**B**) displaying a bright field; cell morphology (left panel) and green fluorescence; and a 2-NBDG signal (middle panel). The fluorescence signal is overlayed on the bright field image to visualize the co-localization of 2-NBDG within the cellular context (right panel). The cellular uptake of 2-NBDG (**C**). Relative fluorescence pixel intensity analyzed by the Zeiss ZEN Pro software (version 3.9). The data are indicated as the mean ± SD of three independent experiments. The differences between the treatment groups were determined using a one-way analysis of variance (ANOVA), followed by Tukey’s multiple comparison. * *p* values < 0.05 and ** *p* values < 0.01 vs. TNF-α-treated control.

**Figure 2:**
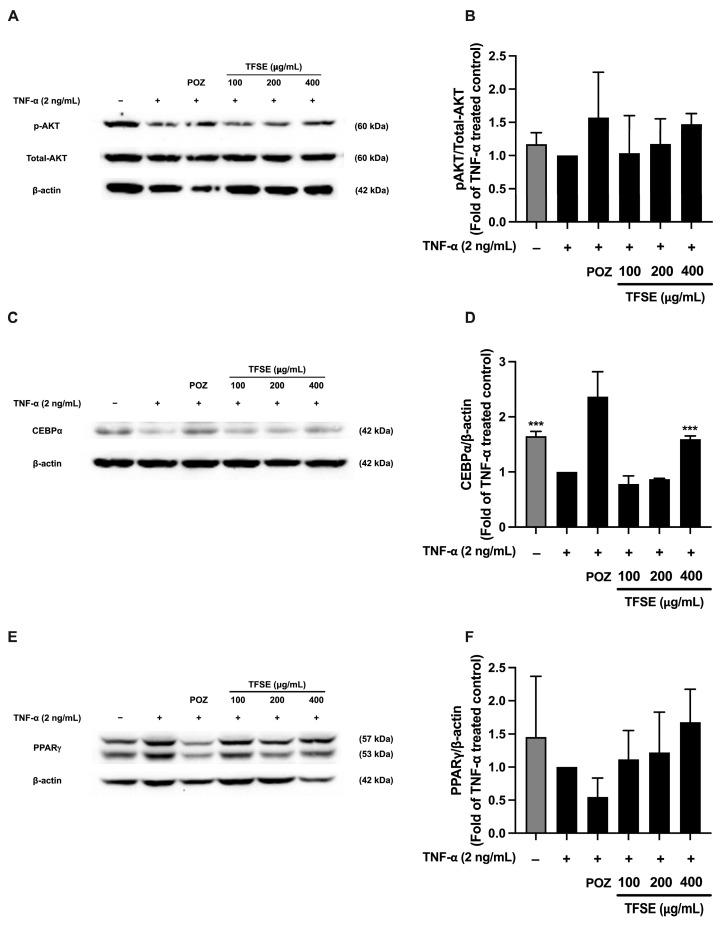
TFSE rescued adipocyte dysfunction, resulting in improved insulin sensitivity. After treatment, the protein samples were collected and used to determine key downstream markers of insulin signaling; the phosphorylation of AKT (pAKT) and adipogenic markers; and PPARγ and CEBPα via Western blotting. The relative phosphorylation levels of Akt were calculated by dividing the amount of protein detected by the phosphorylated antibody by the amount detected by the antibody to determine the total protein levels. The band density of phospho- and total forms of Akt (**A**). The level of the protein phosphor and total forms of Akt (**B**). The band density and the level of the protein CEBPα (**C**,**D**). The band density and the level of the protein PPARγ (**E**,**F**). Western blotting results are shown from three independent experiments. The band density of targeted protein normalized with β-actin levels. Each value in (**B**,**D**,**F**) represents the mean ± SD (n = 3) *** *p* < 0.001 vs. TNF-α-treated control.

Non-toxic concentrations of non-optimized TFSE were used to examine soybean’s inhibitory effect on insulin resistance in 3T3-L1 adipocytes. As shown in Figure 1A, the cell viability of mature 3T3-L1 adipocytes after TFSE treatment was not significantly different from the untreated control, indicating that TFSE up to 400 µg/mL was non-toxic to the cells. Therefore, non-toxic concentrations between 100 and 400 µg/mL were selected for further experiments. The effect of TSFE on inflammation-induced insulin resistance was determined by measuring glucose uptake in TNF-α-treated mature 3T3-L1 adipocytes. As shown in Figure 1B,C, the glucose analog 2-NBDG uptake in the control cells was approximately 2-fold higher than TNF-α-treated 3T3-L1 cells, indicating TNF-α-induced insulin resistance. However, treatment with 400 µg/mL TSFE significantly increased glucose uptake by approximately 2-fold compared to the TNF-α-treated controls. Additionally, TFSE promoted the phosphorylation of AKT, a key insulin signaling mediator, in TNF-α -treated 3T3-L1 cells, though this increase was not statistically significant (Figure 2A,B). Overall, these results suggest that TSFE minimizes inflammation-induced insulin resistance and improves insulin sensitivity in mature adipocytes.

The levels of expression of PPARγ and CEBPα, which are key adipogenic proteins typically found in mature adipocytes, were measured to evaluate adipocyte dysfunction following TNFα-treatment in 3T3-L1 cells. The levels of both proteins decreased after TNFα treatment (Figure 2C–F). Notably, TFSE at 400 µg/mL significantly induced the expression of CEBPα, while the increase in PPARγ was not statistically significant compared to the TNFα-treated group (Figure 2C,D).

These results indicate that TNFα-induced inflammation impaired adipocyte function, but TFSE treatment can partially counteract these negative effects. Specifically, TFSE restored insulin sensitivity, as shown by the significant upregulation of the expression of CEBPα, suggesting enhanced adipogenesis. Although improvements in other markers like PPARγ or p-AKT were observed, they did not reach statistical significance. Thus, these findings suggest that TFSE primarily improves adipocyte function via the upregulation of CEBPα, while its effects on other signaling pathways require further investigation.

### 3.2. Microwave-Assisted Extraction Optimization of TFSE Using Box–Behnken Design (BBD) and Response Surface Methodology (RSM)

A Box–Behnken Design (BBD) in combination with response surface methodology (RSM) was employed to guide and simplify the optimization of the TFSE extraction. Widely used in the food and pharmaceutical industries, BBD and RSM help improve extraction efficiency while reducing resource use, time, and operational costs. Microwave-assisted extraction (MAE) was applied as an environmentally friendly approach, optimizing three parameters: ethanol concentration (% *v*/*v*), microwave power (W), and extraction time (s). Table 3 shows the 15 experimental runs generated via BBD, along with the measured values of TPC, TFC, and ORAC, and specific compounds such as protocatechuic acid, daidzein, genistein, and glycitein. HPLC analysis confirmed the presence of these phenolic and isoflavone compounds in TFSE (Figure S1). As shown in Table 4 and Table 5, the ANOVA results presented statistical significance for models predicting TFC, ORAC, daidzein, genistein, and glycitein, indicating their suitability for determining optimal extraction parameters. Although the optimized extraction conditions resulted in an increased total phenolic content (TPC) in the final extract, the RSM model for TPC was not statistically significant. This could be due to the complexity of extracting phenolics from fermented soybean, possible matrix effects, or nonlinear interactions between extraction variables and TPC. These findings highlight the improvement of using multiple response variables when optimizing the extraction process.

**Figure 3:**
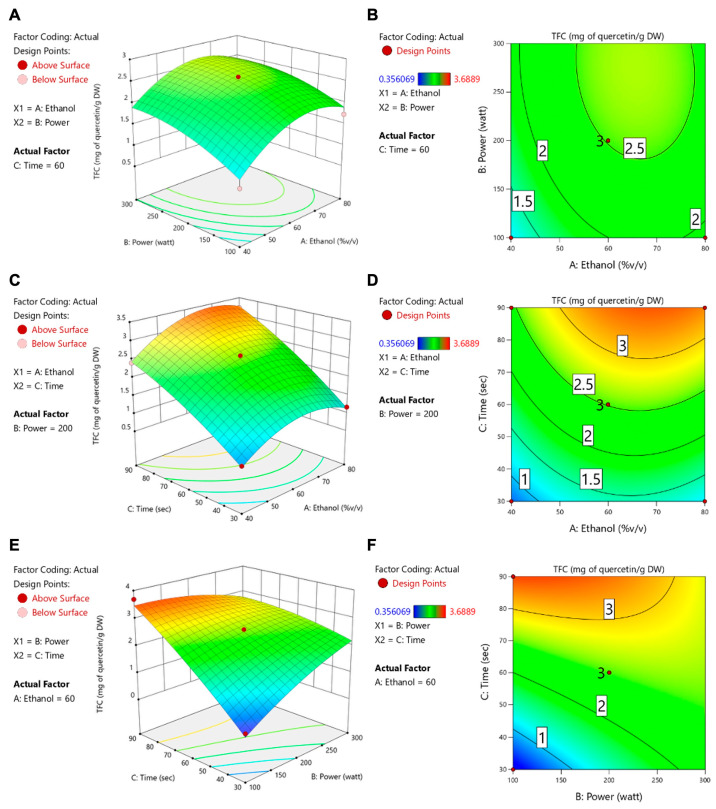
Contour plots (**A**,**C**,**E**) and response surface plots (**B**,**D**,**F**) of TFC affected by ethanol concentration (% *v*/*v*), power (W), and extraction time (s).

**Figure 4:**
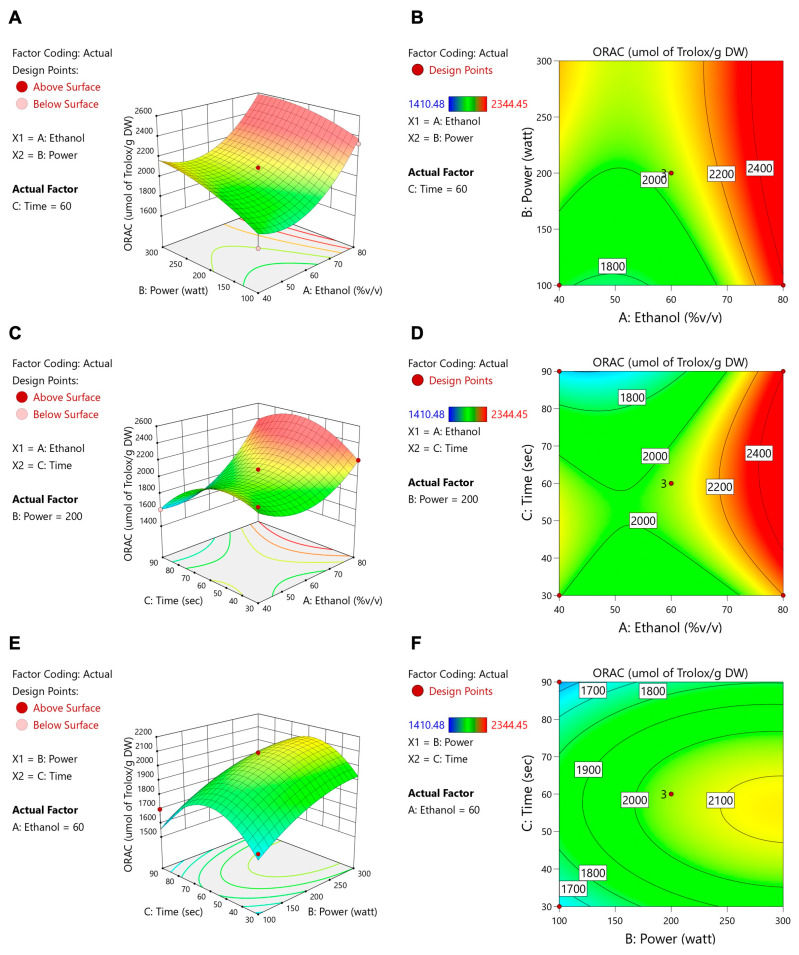
Contour plots (**A**,**C**,**E**) and response surface plots (**B**,**D**,**F**) of ORAC affected by ethanol concentration (% *v*/*v*), power (W), and extraction time (s).

**Figure 5:**
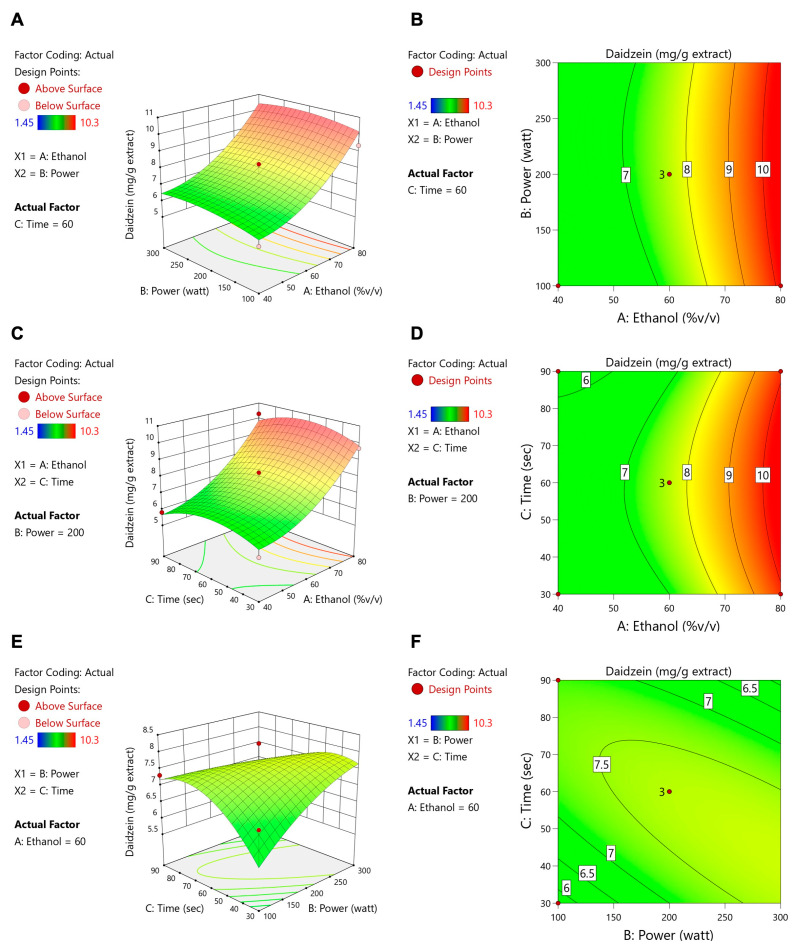
Contour plots (**A**,**C**,**E**) and response surface plots (**B**,**D**,**F**) of daidzein affected by ethanol concentration (% *v*/*v*), power (W), and extraction time (s).

**Figure 6:**
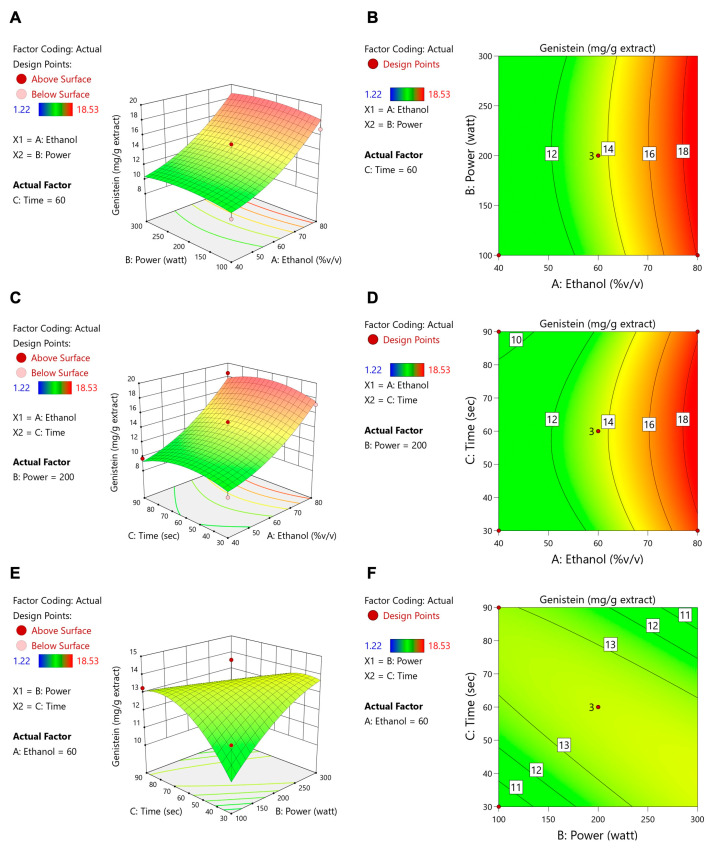
Contour plots (**A**,**C**,**E**) and response surface plots (**B**,**D**,**F**) of genistein affected by ethanol concentration (% *v*/*v*), power (W), and extraction time (s).

**Figure 7:**
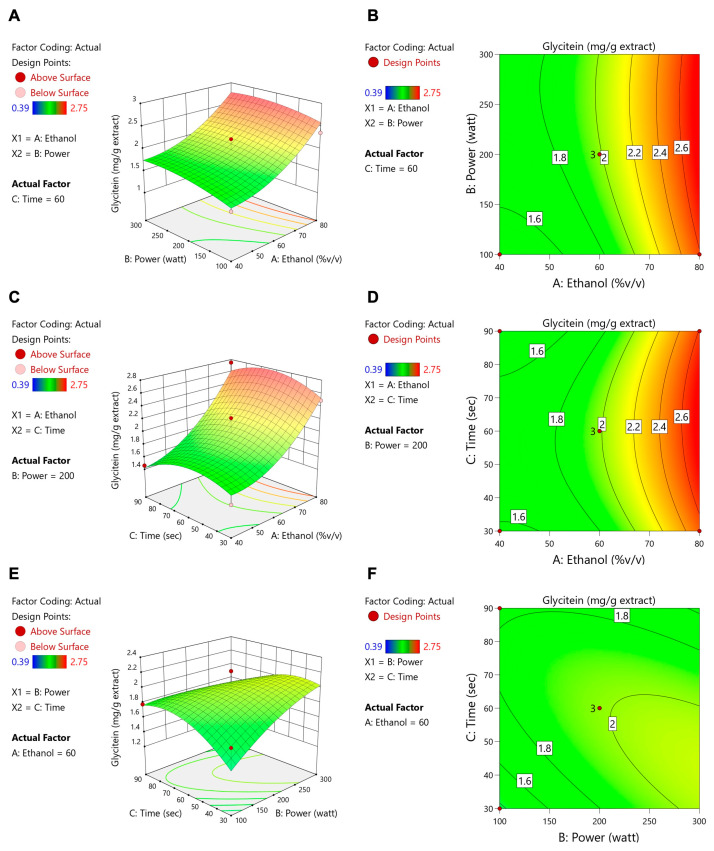
Contour plots (**A**,**C**,**E**) and response surface plots (**B**,**D**,**F**) of glycitein affected by ethanol concentration (% *v*/*v*), power (W), and extraction time (s).

Table 4 shows that ethanol concentration, microwave power, and extraction time all had significant impacts on the total flavonoid content (TFC), whereas only the ethanol concentration significantly affected ORAC and isoflavone levels (daidzein, genistein, and glycitein). These data align with the contour and response surface plots (Figure 3, Figure 4, Figure 5, Figure 6 and Figure 7). For TFC, both ethanol concentration and microwave power had significant positive effects, with TFC rising as ethanol reached about 60–70% (*v*/*v*) and microwave power increased to 400–500 W. However, further increases in these parameters led to a plateau or slight decline in TFC, likely due to solvent saturation or the thermal degradation of flavonoids. The extraction time also influenced TFC, with longer durations enhancing yields at lower ethanol concentrations but offering diminishing returns at higher concentrations.

With regard to antioxidant activity (ORAC assay, Figure 4), the ethanol concentration was again the most influential factor, with maximum activity being observed at 60–70% ethanol. The moderate levels of microwave power and extraction time provided further improvements, whereas excessive heat or prolonged extraction could reduce antioxidant effectiveness. Regarding isoflavone compounds (daidzein, genistein, and glycitein), the response surface plots (Figure 5, Figure 6 and Figure 7) revealed that lower microwave power and shorter extraction times were most beneficial, as these compounds are heat-sensitive. Altogether, the response surface analysis emphasized the need to precisely balance the ethanol concentration, microwave power, and extraction time to achieve optimal yields of flavonoids and antioxidants while protecting heat-sensitive isoflavones. These results highlight the importance of statistical levels of optimization for refining extraction processes in the production of functional food ingredients.

**Figure 8:**
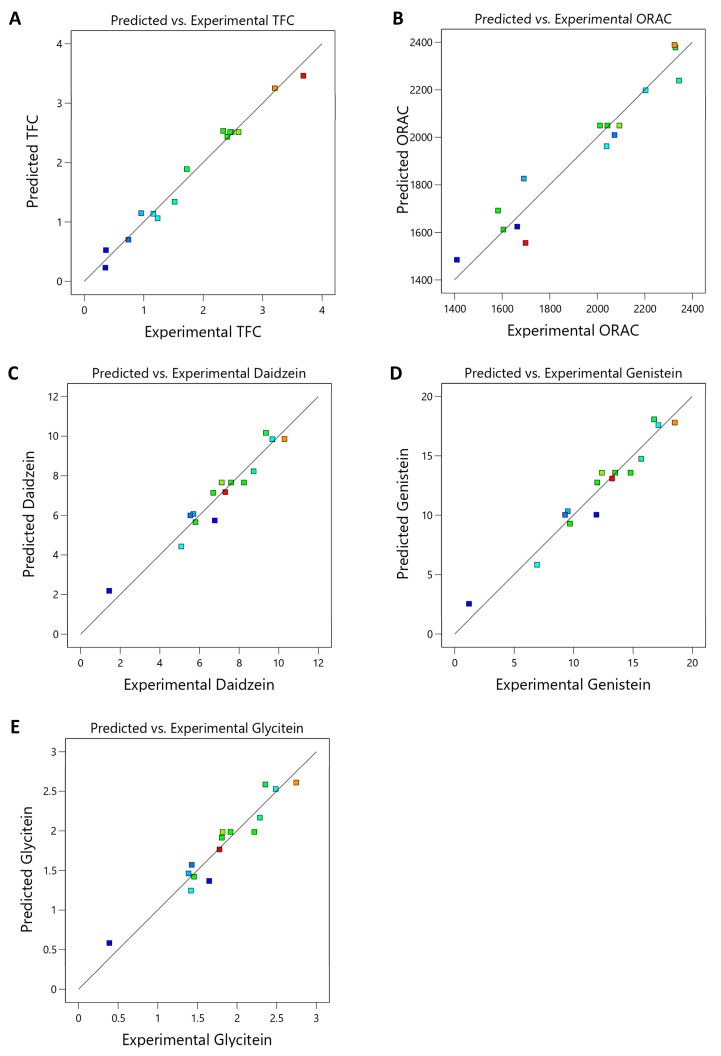
Experimentally and predicted measures of TFC (**A**), ORAC (**B**), daidzein (**C**), genistein (**D**), and glycetein (**E**) calculated from Equations (2)–(6).

The plots comparing predicted and experimental values for TFC, ORAC, daidzein, genistein, and glycitein showed exponential curves (Figure 8), indicating a strong correlation for each outcome. The high correlation was supported by the R^2^ values in Table 4 and Table 5, all exceeding 0.9 for these variables. The ANOVA analysis for the optimization equations is presented as Equations (2)–(6), which can be used to guide the selection of optimal extraction parameters; however, their level of accuracy needs to be validated experimentally. Additionally, scaling up the process will require further consideration of operational and practical factors beyond those identified at the laboratory level. According to Design-Expert® software (DX13), Version 13, the optimal microwave extraction conditions for maximizing TFC, ORAC, protocatechuic acid, glycitein, genistein, and daidzein were 80% EtOH, 100 W microwave power, and 80 s of extraction time. Under these conditions, the desirability value was 0.954, indicating a high level of suitability for the extraction process. In comparison to the conventional method, which involved soaking Thua Noa powder in 80% EtOH for 12 h three times for a total of 36 h, the optimized approach reduced the extraction time to just 80 s. These findings demonstrate the effectiveness of using BBD and RSM for process optimization, and this optimized condition was used in the subsequent experimental step.

**Equation for Variable Optimization**

TFCs = −9.088 + 0.1556A + 0.026B + 0.086C − 0.00003AB + 0.00016AC − 0.0002BC − 0.0012A^2^ − 0.00002B^2^ − 0.0002C^2^ (2)

ORAC = 2786.248 − 76.404A + 5.482B + 18.687C − 0.017AB + 0.245AC − 0.0058BC + 0.6433A^2^ − 0.0068B^2^ − 0.2832C^2^ (3)

Glycitein = 0.556806 − 0.051903A + 0.010166B + 0.035537C − 0.000013AB + 0.000096AC − 0.000072BC + 0.000629A^2^ − 9.79861E-06B^2^ − 0.000229C^2^ (4)

Genistein = 2.07139 − 0.248250A + 0.053411B + 0.251796C + 0.000074AB + 0.000400AC − 0.000553BC + 0.003419A^2^ − 0.000057B^2^ − 0.001414C^2^ (5)

Daidzein = 2.68042 − 0.180403A + 0.030790B + 0.140667C − 0.000019AB + 0.000146AC − 0.000266BC + 0.02300A^2^ − 0.000030B^2^ − 0.000825C^2^ (6)

where A, B, and C are the independent variables, including ethanol concentration (% *v*/*v*), extraction power (W), and extraction time (s), respectively.

### 3.3. The O-TFSE and TFSE Exhibited Distinctive Levels of Phytochemicals

Phytochemicals are well known for their health-promoting properties, particularly due to their ability to inhibit key enzymes involved in the progression of various human diseases [52]. Such enzymes are, therefore, frequent targets for clinically approved drugs. In this study, the metabolomic profiles of chemicals in both O-TFSE and TFSE were analyzed using HPLC-qTOF/MS in the positive ion mode. Only compounds with library scores above 95% were included in Tables S1 and S2 to ensure their accuracy. The findings showed that 58 compounds were identified in O-TFSE, while 43 compounds were found in TFSE. Both extracts were composed of amino acids, fatty acid derivatives, nucleotides, indoles, phenolics, flavonoids, and isoflavones, reflecting the complexity of their chemical composition. Notably, phytochemicals such as daidzein, genistein, and glycitein were present at high amounts. Additionally, 1-monolinoleoyl-rac-glycerol and monolinolenin—metabolites of linoleic and linolenic acids—were detected at levels about 10 times higher in O-TFSE in comparison to TFSE.

The quantitative analysis of TPC and TFC for both TFSE and O-TFSE is presented in Table 6. The O-TFSE, produced via MAE with 80% ethanol, 100 W, and 80 s, contained significantly higher amounts of phytochemicals than TFSE extracted by ethanol alone (*p* < 0.0001). The TFC of O-TFSE reached 3.16 ± 0.01 mg QE/g DW, which is 31 times higher than TFSE (0.11 ± 0.00 mg QE/g DW). While the optimized extraction condition did not significantly predict TPC as previously noted, the TPC of O-TFSE was 45.36 ± 0.62 mg GAE/g DW, which is almost 22 times higher than the TPC of TFSE (2.06 ± 0.02 mg GAE/g DW), highlighting the effectiveness of the optimized method.

HPLC analysis (Table 6) revealed that the concentrations of key isoflavones, including daidzein, genistein, and glycitein, were lower in O-TFSE compared to TFSE. Specifically, O-TFSE contained 12.8 ± 0.03 mg/g daidzein, 23.4 ± 0.19 mg/g genistein, and 3.2 ± 0.00 mg/g glycitein, which were 6-, 5-, and 2 times lower, respectively, than those detected in TFSE (72.8 ± 0.04, 107.4 ± 0.24, and 6.8 ± 0.01 mg/g extract).

### 3.4. Optimized TFSE (O-TFSE) Demonstrated Superior Antioxidant Activity Compared to TFSE Derived from Conventional Extraction

Antioxidants are capable of neutralizing free radicals through both single electron transfer (SET) and hydrogen atom transfer (HAT) mechanisms. Since oxidative stress is a key contributor to the development of insulin resistance and type 2 diabetes mellitus (T2DM), antioxidant activity was assessed in both O-TFSE and TFSE. Reactive oxygen species (ROS), which are produced during metabolic dysfunction, can disrupt insulin signaling, damage cellular components, and promote inflammation, ultimately leading to adipocyte dysfunction and glucose intolerance [20,21]. Therefore, the assessment of the antioxidant capacity of these extracts provides important insights into their potential to counteract oxidative stress and support cellular health.

In this study, the antioxidant activity of O-TFSE and TFSE was mainly mediated via the HAT pathway, as evidenced by higher ORAC values in comparison to DPPH and FRAP values (markers of the SET mechanism), as shown in Table 6. Nonetheless, some involvement of the SET pathway was also observed, indicating that both mechanisms play a role, with HAT being predominant [53]. ORAC, FRAP, and DPPH assays showed that O-TFSE exhibited much higher antioxidant activities, with values of 2613.75 ± 26.60, 44.93 ± 2.19, and 18.12 ± 1.21 µmol TE/g DW, respectively, in comparison to TFSE (110.78 ± 1.44, 1.995 ± 0.10, and 1.093 ± 0.04 µmol TE/g DW, all *p* < 0.0001). These results confirm that the optimized method significantly enhanced the antioxidant capacity of the extract. This improvement is likely due to the significant increase in phenolic and flavonoid contents achieved through optimization.

### 3.5. Optimized TFSE (O-TFSE) Exhibited Higher Inhibitory Effect on α-Glucosidase and α-Amylase Compared to TFSE Obtained from Conventional Extraction

O-TFSE exhibited substantially stronger inhibitory effects on carbohydrate-hydrolyzing enzymes, α-glucosidase and α-amylase, than TFSE (Table 7). At 6.25 mg/mL, O-TFSE inhibited α-glucosidase by 72.91 ± 1.19%, which is 1.2 times higher than TFSE (59.77 ± 1.37%). Similarly, O-TFSE showed a 64.31 ± 1.44% inhibition of α-amylase, in comparison to 37.09 ± 0.90% for TFSE (*p* < 0.0001). These findings highlight the effectiveness of the eco-friendly MAE technique in greatly improving both the phytochemical content and the functional bioactivities of the extract, particularly in terms of antioxidant and anti-diabetic properties.

### 3.6. Molecular Docking and Molecular Dynamics (MDs) Simulations

**Figure 9:**
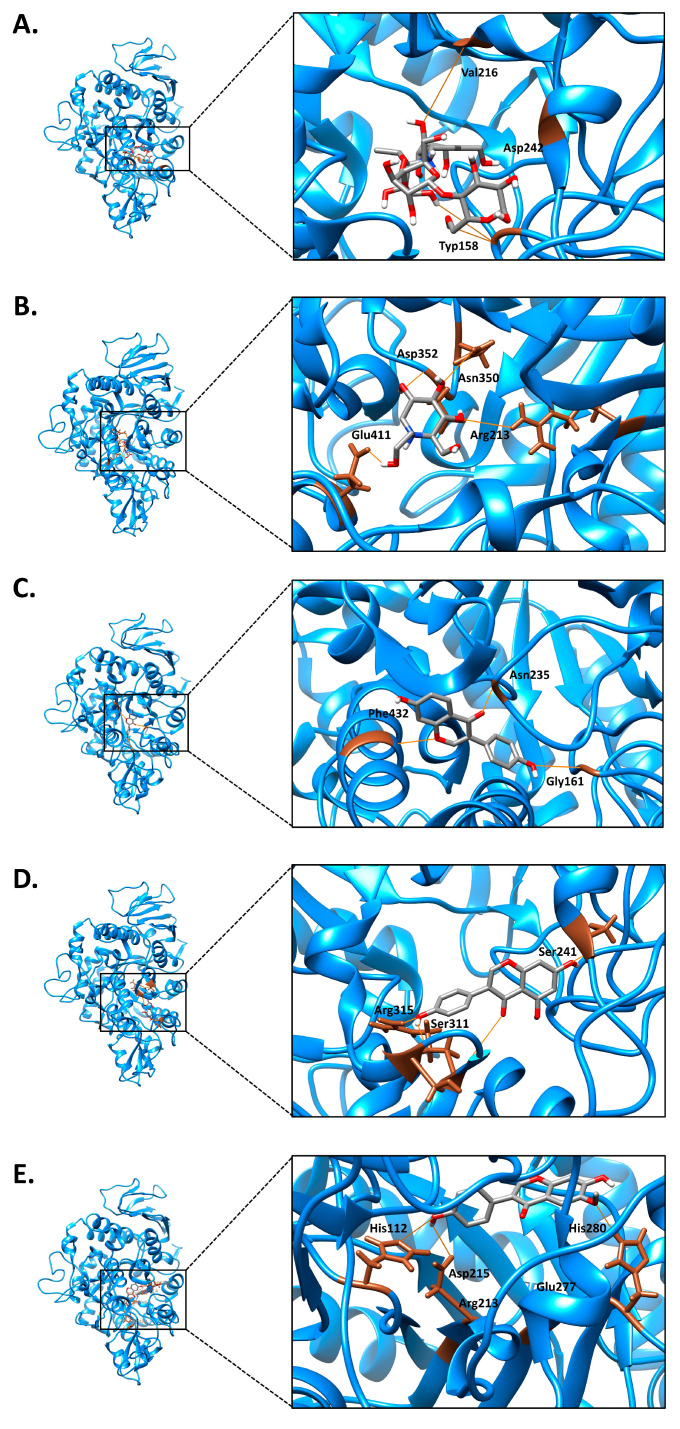
The 3D structural prediction of each chemical compound on *Saccharomyces cerevisiae* α-glucosidase (PDB: 3A4A). (**A**) Acarbose and the interactions on the active site; (**B**) miglitol and the interactions on the active site; (**C**) daidzein and the interactions on the active site; (**D**) genistein and the interactions on the active site; (**E**) glycitein and the interactions on the active site.

O-TFSE demonstrated promising anti-diabetic activity, particularly by inhibiting α-glucosidase (Table 7), suggesting its potential as an anti-diabetic agent. This effect was further explored using molecular docking and molecular dynamics (MDs) simulations to study how O-TFSE isoflavones interact with the active site of α-glucosidase. Figure 9 illustrates the binding interactions between the top three isoflavones in O-TFSE (daidzein, genistein, and glycitein) as well as the reference drugs acarbose and miglitol. Table 8 summarizes the binding energies and the specific amino acid residues involved. The results showed that miglitol interacted with more amino acid residues than acarbose, though acarbose had a lower binding energy (−8.94 kcal/mol), indicating a more stable and energetically favorable binding. This was attributed to acarbose forming stronger or more specific interactions such as hydrogen bonds or electrostatic contacts with key residues in the enzyme’s active site. In contrast, miglitol formed more but weaker interactions. Daidzein (−8.55 kcal/mol), genistein (−8.36 kcal/mol), and glycitein (−8.41 kcal/mol) all had binding energies close to that of acarbose (−8.94 kcal/mol), although they interacted with different amino acid residues. This suggests that these isoflavones may have significant inhibitory potential against α-glucosidase, supporting the anti-diabetic promise of O-TFSE.

**Figure 10:**
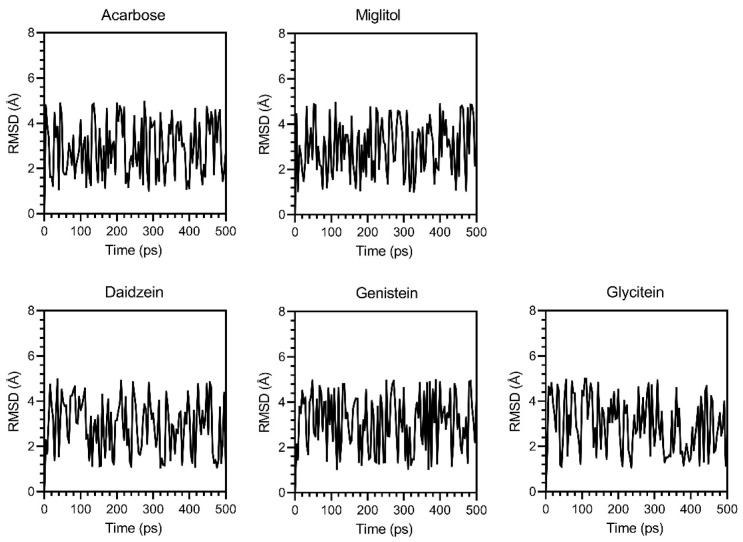
Time evolution of the backbone root mean square deviation (RMSD) of α-glucosidase upon binding to acarbose, miglitol, daidzein, genistein, and glycitein during a 500 ps MD simulation.

**Figure 11:**
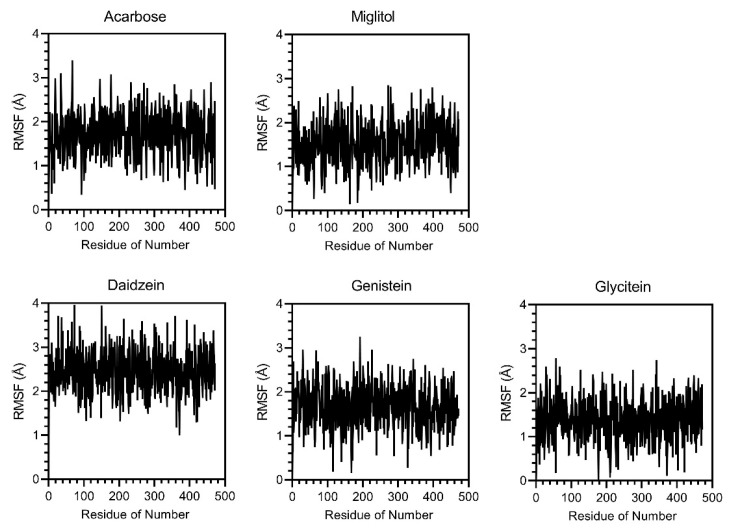
Per-residue root mean square fluctuation (RMSF) profiles of α-glucosidase in complex with acarbose, miglitol, daidzein, genistein, and glycitein over the course of a 500 ps and 472 residue MD simulation.

**Figure 12:**
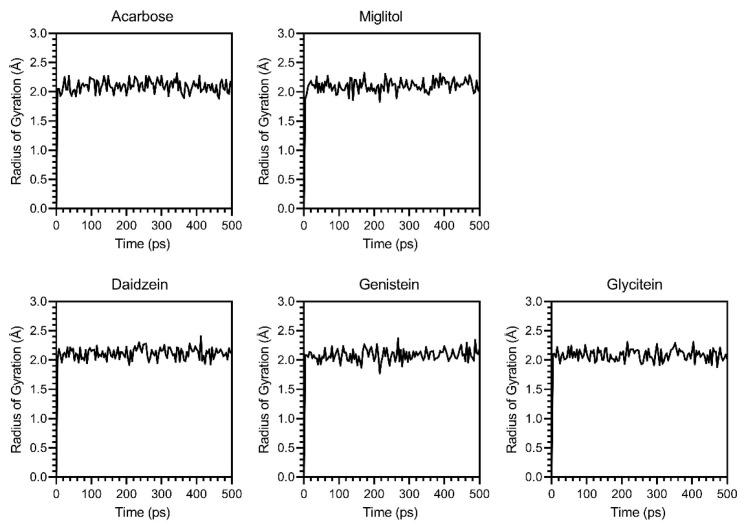
Radius of gyration (Rg) analysis of α-glucosidase–ligand complexes with acarbose, miglitol, daidzein, genistein, and glycitein to assess global structural compactness throughout a 500 ps MD simulation.

While molecular docking only provides a static snapshot of ligand–protein interactions, molecular dynamics (MDs) simulations were used to validate the dynamic interactions between ligands and α-glucosidase. Three parameters, RMSD, RMSF, and Rg, were analyzed over a simulation of 500 ps. The RMSD score fluctuated between 1.0 and 5.0 Å (Figure 10) during the simulation, indicating moderate stability, with all tested compounds remaining bound to the active site. RMSF results (Figure 11) showed that genistein and glycitein had low and uniform fluctuations (0.18 to 3.24 Å and 0.01 to 2.766 Å, respectively), suggesting stable protein conformations. In contrast, daidzein induced higher flexibility (0.8 to 4 Å), suggesting a weaker or less stabilizing interaction. Analysis of the Rg scores (Figure 12) demonstrated that α-glucosidase remained at a low and stable radius of gyration (1.88 and 2.32 Å) across all ligand–protein complexes, indicating strong overall structural integrity during the simulation period. These MD results confirmed that daidzein, genistein, glycitein, acarbose, and miglitol all interacted with α-glucosidase without disrupting its structure. Among them, glycitein was the most stable binder, as evidenced by its ability to maintain the most compact and consistent protein conformation.

### 3.7. Optimized TSFE and Its Active Isoflavones Suppress Inflammation-Induced Insulin Resistance in TNF-α-Treated 3T3-L1 Adipocytes

**Figure 13:**
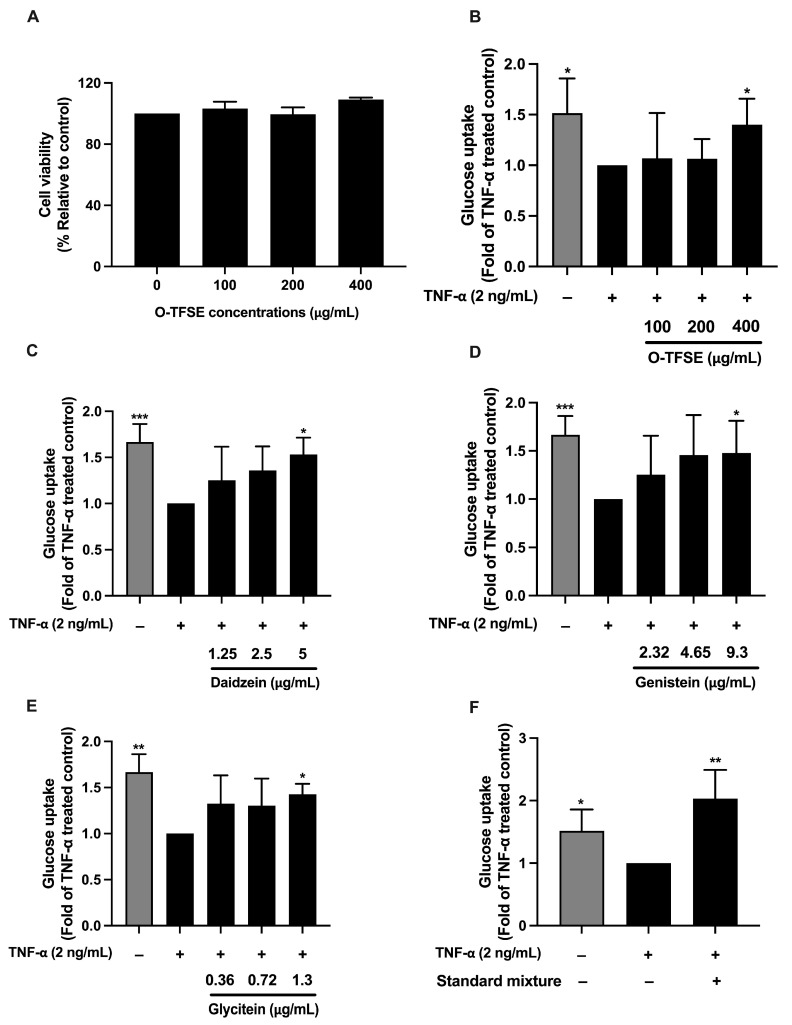
The cytotoxicity of O-TFSE in 3T3-L1 adipocytes as determined by the SRB assay (**A**). The cells were treated with various doses of O-TFSE (12.5–400 µg/mL) for 72 h. The effect of O-TFSE and the isoflavones on insulin-induced cellular glucose uptake in TNF-α-treated 3T3-L1 adipocytes (**B**–**F**). The effect of O-TFSE (**B**), daidzein (**C**), genistein (**D**), glycitein (**E**), and a mixture of the isoflavone standards (5 µg/mL daidzein, 9.3 µg/mL genistein, and 1.3 µg/mL glycitein) (**F**) on the cellular uptake of 2-NBDG. Relative fluorescence pixel intensity analyzed by the Zeiss ZEN Pro software (version 3.9). The data are shown as the mean ± SD of three independent experiments. The differences among the treatment groups were determined using a one-way analysis of variance (ANOVA), followed by Tukey’s multiple comparison. * *p* Values < 0.05, and ** *p* Values < 0.01, *** *p* Values < 0.001 were regarded as a measure of statistical significance.

Non-toxic doses of O-TFSE were used to evaluate both its own and its major isoflavones’ ability to counteract insulin resistance in 3T3-L1 adipocytes. As shown in Figure 13A, O-TFSE at concentrations up to 400 µg/mL did not reduce cell viability compared to untreated controls, confirming its safety for further use in the 100–400 µg/mL range. To assess the effect on inflammation-induced insulin resistance, glucose uptake was measured in TNF-α-treated mature 3T3-L1 adipocytes. As shown in Figure 13B, TNF-α treatment significantly reduced glucose analog (2-NBDG) uptake, showing about a 1.5-fold decrease compared to untreated cells. In contrast, O-TFSE treatment at 400 µg/mL led to a 1.4-fold increase in glucose uptake versus TNF-α-treated controls.

Further analysis evaluated the insulin-sensitizing activities of daidzein, genistein, and glycitein at concentrations equivalent to their levels in 400 µg/mL O-TFSE, which were evaluated by a glucose uptake assay, using a comparable quantity detected in O-TFSE (5, 9.3, and 1.3 µg/mL, respectively). Each compound significantly increased glucose uptake by 1.5-, 1.4-, and 1.4-times, respectively, in comparison to the TNF-α-treated control (Figure 13B–D). Testing a combination of all three isoflavones at these same concentrations revealed an even more pronounced effect, doubling the cellular absorption of 2NBDG-glucose in comparison to TNF-α-treated cells (Figure 13E). This suggests a potential synergistic benefit when these isoflavones are present together, as seen in the extracts.

## 4. Discussion

This study successfully optimized the microwave-assisted extraction (MAE) of bioactive compounds from Thua-Nao, a traditional Thai fermented soybean (TFS) product, highlighting both technological improvements and the health-promoting potential of Thua-Nao as a functional food ingredient for diabetes prevention.

Using a Box–Behnken design (BBD) and response surface methodology (RSM), this study systematically determined the best extraction conditions (80% ethanol, 100 W, and 80 s), which produced extracts with much higher levels of phenolics, flavonoids, antioxidant activity, α-amylase, and α-glucosidase inhibition in comparison to conventional methods. This was mainly due to efficient cell wall disruption and improved solvent penetration using aqueous ethanol [54,55,56]. These results were consistent with previous studies that emphasized the importance of optimizing solvent polarity and microwave power for the effective extraction of phenolic compounds from plant matrices [57,58]. Ethanol, as a green solvent, is well-suited for food and pharmaceutical applications. Microwave technology accelerates heating and enhances the solvent’s access to the plant matrix, thereby reducing extraction time and energy consumption. This approach has been supported by numerous studies for the extraction of bioactive compounds from various plant sources including soybeans [58,59,60].

The enhanced antioxidant activity observed in the optimized extract is particularly relevant for diabetes management, as strong antioxidants can help restore redox balance, protect pancreatic β-cells, and improve insulin sensitivity [20,21,22,23]. The optimized extract had notably higher phenolic and flavonoid contents, which are known to scavenge reactive oxygen species (ROS) and protect cells from oxidative damage. Polyphenols are known for their ability to neutralize ROS and protect cellular components from oxidative damage [56,61,62]. The robust antioxidant capacity of O-TFSE suggests that it is promising as a functional food to help prevent oxidative stress-related conditions, such as diabetes, heart disease, and some cancers.

The inhibitory effects of O-TFSE and TFSE on α-glucosidase and α-amylase, key enzymes in carbohydrate digestion, were subsequently assessed. The inhibition of these enzymes delays glucose absorption and lowers postprandial blood glucose levels, which are beneficial for diabetes management [63,64,65]. O-TFSE demonstrates stronger inhibition than TFSE, likely due to its higher total phenolic and flavonoid contents. These compounds can act together with isoflavones (such as daidzein, genistein, and glycitein), binding to the active sites of the enzymes and enhancing inhibitory effects, even though individual isoflavone concentrations were higher in TFSE. This suggests that the combined phytochemical profile—especially polyphenols and flavonoids—plays a crucial role in enzyme inhibition.

The differences in the metabolite profiles between O-TFSE (optimized extract) and TFSE (non-optimized extract) are mainly attributable to the extraction techniques used. Microwave-assisted extraction (MAE) rapidly and evenly heats the material, breaking down cell walls more efficiently and releasing a broader spectrum and higher amounts of phytochemicals. As a result, more compounds were identified in O-TFSE (58) in comparison to TFSE (43).

Significantly elevated levels of specific metabolites such as 1-monolinoleoyl-rac-glycerol and monolinolenin—which is up to 10 times higher in O-TFSE—suggest that MAE is particularly effective for the extraction of certain lipid-derived and thermolabile compounds, as well as those less soluble in standard conditions. Improved solvent penetration and enhanced mass transfer during MAE can also help release both free and bound phytochemicals that are not easily accessed by conventional extraction.

Furthermore, differences in the abundance of key phytochemicals, including daidzein, genistein, and glycitein, may result from the selective extraction power of MAE or the possible degradation or transformation of compounds under varying extraction conditions. In summary, the optimized extraction not only accelerated the process but also enhanced the diversity and potential bioactivity of the phytochemical profile.

Metabolomic profiling and HPLC analyses revealed that both non-optimized and optimized TFS extracts (TFSE and O-TFSE) contained high levels of daidzein, genistein, and glycitein, which are well-known bioactive isoflavones with estrogenic and antioxidant activities [66]. This abundance emphasized their potential as sources of functional ingredients. These isoflavones have been extensively linked to reduced oxidative stress, improved lipid metabolism, and enhanced insulin sensitivity [67,68,69]. Although O-TFSE showed lower isoflavone levels than TFSE, its antioxidant and enzymatic inhibitory activities (against α-glucosidase and α-amylase) surpassed those of TFSE. This suggests that the synergistic effects of multiple phytochemicals, including phenolics, flavonoids, and other metabolites in O-TFSE, play a key role in this extract’s enhanced efficacy. Previous research established soybeans as a rich source of diverse bioactive compounds, including peptides, saponins, and other flavonoids such as isoflavones. Together, these constituents exert beneficial effects on glucose metabolism and insulin sensitivity, often exhibiting synergistic effects. In particular, soy peptides and saponins demonstrate insulin-sensitizing and anti-inflammatory properties, which compensate for the reduced isoflavone content in O-TFSE and contribute to its preserved biological activity [70].

Among the metabolites, 1-monolinoleoyl-rac-glycerol and monolinolenin—monoglyceride metabolites derived from linoleic acid and α-linolenic acid, respectively—were detected in O-TFSE at levels tenfold higher than in TFSE. 1-Monolinoleoyl-rac-glycerol is primarily recognized for its antiviral activity, while monolinolenin demonstrates potent antibacterial effects through the disruption of bacterial membranes. Both compounds are naturally present in certain fermented foods, highlighting the diverse functional roles of fatty acid-derived monoglycerides in biological systems. Their elevated presence in O-TFSE suggests that they may contribute to the observed biological activities in this study. However, further research is warranted to determine whether these metabolites also possess anti-diabetic properties [71,72,73].

One of the most compelling findings in recent insulin resistance research involves experimental models using TNF-α-treated 3T3-L1 adipocytes. Studies have demonstrated that TFSE significantly increases insulin-induced glucose uptake in cells, indicating improved insulin sensitivity [74]. This effect shows a correlation with enhanced phosphorylation of AKT, a critical downstream effector in the insulin signaling pathway, and is essential for GLUT4 transporter translocation and subsequent cellular glucose uptake [74]. The upregulation of C/EBPα and PPARγ protein levels contributes to improved adipocyte function by activating key metabolic genes [75]. Such mechanisms show promise for the potential of developing preventive and management strategies for type 2 diabetes [76].

Interestingly, even though O-TFSE had a lower isoflavone content than TFSE, it displayed similar or even superior effects on insulin sensitivity. This improvement is likely due to the extraction optimization process, especially in the case of microwave-assisted extraction (MAE) and possibly ultrasonication, which increased total phenolic and flavonoid content (TPC/TFC) and overall antioxidant capacity, despite reducing certain isoflavones. This trade-off suggests that although some bioactive isoflavones decreased, the increase in other antioxidant compounds in O-TFSE created a synergistic benefit that improved insulin sensitivity. These findings agree with earlier studies showing that advanced extraction techniques, such as MAE and ultrasonication, boost the yield of phenolic antioxidants and enhance the functional properties of plant extracts [77]. The pronounced increase in antioxidant activity in O-TFSE, corresponding with higher TPC and TFC levels, supports its role as a potent functional ingredient. Improved antioxidant capacity is particularly important for the reduction in oxidative stress and inflammation, which are key contributors to insulin resistance [78]. Finally, these findings highlight the necessity of optimizing extraction methods to maximize specific phytochemicals and achieve a well-balanced phytochemical profile and superior biological effects. Future studies should focus on refining extraction protocols through repeated extraction cycles and combining techniques to further maximize the functional properties of these extracts for nutraceutical and therapeutic applications.

## 5. Conclusions

This study successfully optimized the microwave-assisted extraction (MAE) process for the isolation of bioactive compounds from Thua-Nao. In comparison to the conventional method, which required 36 h of extraction, this process enabled a significant reduction in extraction time to just 80 s and resulted in higher concentrations of phenolic and flavonoid compounds, greater antioxidant activity, and stronger inhibition of α-amylase and α-glucosidase. The optimized extract (O-TFSE) demonstrated improved insulin sensitivity in cellular models, likely due to synergistic effects among various phytochemicals, despite reduced isoflavone levels. While MAE boosted phenolics and antioxidants, the decrease in certain isoflavones highlights the importance of balancing bioactive components for targeted health benefits. These findings suggest that the MAE-optimized Thua-Nao extract is a promising, sustainable functional food ingredient for diabetes prevention, or at the very least amelioration. Nonetheless, since the biological effects were only demonstrated in vitro, further in vivo and clinical studies are needed to confirm efficacy and safety in humans. Future studies should also look at integrating MAE with other extraction techniques to further maximize the yield and bioactivity of functional compounds.

## Figures and Tables

**Table 1:** Box–Behnken design matrix with independent variables and actual levels.

Factors	Unit	Actual Levels		
		−1	0	1
(X_1_) Ethanol concentration	% *v*/*v*	40	60	80
(X_2_) Microwave power	W	100	200	500
(X_3_) Extraction time	sec	30	60	90

**Table 2:** The total of actual running experiments derived from BBD.

Run	X_1_: Ethanol Concentration (% *v*/*v*)	X_2_: Extraction Power (W)	X_3_: Extraction Time (sec)
1	60	100	30
2	80	100	60
3	40	100	60
4	60	100	90
5	40	200	90
6	60	200	60
7	60	200	60
8	80	200	30
9	40	200	30
10	60	200	60
11	80	200	90
12	60	500	30
13	80	500	60
14	40	500	60
15	60	500	90

**Table 3:** Coded independent variables (ethanol concentration, power, and time) and dependent variables (TPC, TFC, ORAC, protocatechuic acid, daidzein, genistein, and glycitein) of TFSE derived from the Box–Behnken design (BBD).

Run	Factor 1	Factor 2	Factor 3	Response 1	Response 2	Response 3	Response 4	Response 5	Response 6	Response 7
	A: Ethanol (%*v*/*v*)	B: Power (Watt)	C: Time (s)	TPCs (mg of GAE/g DW)	TFCs (mg of QE/g DW)	ORAC (µmol of Trolox/g DW)	Protocatechuic Acid (mg/g Extract)	Daidzein (mg/g Extract)	Genistein (mg/g Extract)	Glycitein (mg/g Extract)
1	60	100	30	61.57	0.36	1663.95	3.97	6.78	11.94	1.65
2	80	100	60	56.07	1.73	2329.63	4.64	9.36	16.8	2.36
3	40	100	60	52.82	0.96	1691.65	3.88	5.70	9.53	1.39
4	60	100	90	52.86	3.69	1699.22	4.18	7.31	13.26	1.78
5	40	200	90	54.53	2.41	1606.04	3.82	5.81	9.71	1.46
6	60	200	60	60.85	2.48	2012.04	4.49	7.6	13.52	1.92
7	60	200	60	59.13	2.60	2093.82	4.18	7.13	12.41	1.82
8	80	200	30	58.60	1.16	2203.88	4.55	9.69	17.16	2.49
9	40	200	30	52.38	0.74	2073.23	3.82	5.55	9.3	1.43
10	60	200	60	58.01	2.46	2043.39	4.67	8.25	14.81	2.22
11	80	200	90	61.76	3.21	2325.70	5.48	10.3	18.53	2.75
12	60	500	30	56.56	2.34	1583.18	3.98	6.7	12.01	1.81
13	80	500	60	64.85	1.52	2344.45	4.69	8.74	15.71	2.29
14	40	500	60	61.28	1.24	2039.74	3.97	5.09	6.95	1.42
15	60	500	90	52.57	0.37	1410.48	3.07	1.45	1.22	0.39

GAE: gallic acid equivalent; QE: quercetin equivalent; DW: dry weight.

**Table 4:** Analysis of variance, regression coefficients, and *p* value of the second-order polynomial models for TFC and ORAC.

Source	TFCs				ORAC			
	Coefficient	F-Value	*p* Value	Significant	Coefficient	F-Value	*p* Value	Significant
Model	−2.51	29.21	0.0008	***	2049.75	7.69	0.0185	*
A-Ethanol	0.3129	13.02	0.0154	*	241.36	23.64	0.0046	**
B-Power	0.3165	8.93	0.0305	*	136.44	5.06	0.0742	
C-Time	0.9610	122.78	0.0001	***	−51.68	1.08	0.3455	
AB	−0.0580	1.12	0.3388		−34.46	1.20	0.3224	
AC	0.0950	0.6671	0.4512		147.25	4.89	0.0780	
BC	−0.6548	142.51	<0.0001	****	−17.33	0.3047	0.6047	
A^2^	−0.4790	15.64	0.0108	*	257.31	13.78	0.0138	*
B^2^	−0.1979	21.53	0.0056	**	−68.43	7.86	0.0378	*
C^2^	−0.1519	1.57	0.2653		−254.85	13.51	0.0144	*
Residual								
Lack of Fit		15.55	0.0610	NS		16.71	0.0570	NS
R^2^			0.9813				0.9326	
Adjusted R^2^			0.9477				0.8112	

Statistical analyses were determined by one-way analysis of variance (ANOVA). *, *p* < 0.05; **, *p* < 0.01; ***, *p* < 0.001; ****, *p* < 0.0001; NS: not statistically significant.

**Table 5:** Analysis of variance, regression coefficients, and *p* value of the second-order polynomial models for isoflavone compounds (daidzein, genistein, and glycitein).

Source	Daidzein				Genistein				Glycitein			
	Coefficient	F-Value	*p* Value	Significant	Coefficient	F-Value	*p* Value	Significant	Coefficient	F-Value	*p* Value	Significant
Model	7.66	8.08	0.0166	*	13.58	9.94	0.0105	*	1.99	6.71	0.0248	*
A-Ethanol	2.01	33.04	0.0022	**	4.01	39.45	0.0015	**	0.5364	28.84	0.0030	**
B-Power	0.1581	0.1369	0.7265		0.1708	0.0479	0.8355		0.1166	0.9134	0.3831	
C-Time	−0.0825	0.0556	0.8230		−0.1322	0.0428	0.8443		−0.0169	0.0288	0.8719	
AB	−0.0378	0.0291	0.8711		0.1475	0.1331	0.7302		−0.0253	0.1601	0.7056	
AC	0.0875	0.0347	0.8595		0.2400	0.0783	0.7908		0.0575	0.1841	0.6857	
BC	−0.7975	12.99	0.0155	*	−1.66	16.82	0.0093	**	−0.2161	11.70	0.0188	*
A^2^	0.9200	3.55	0.1184		1.37	2.35	0.1861		0.2517	3.26	0.1310	
B^2^	−0.3031	3.11	0.1383		−0.5742	3.34	0.1272		−0.0980	3.98	0.1025	
C^2^	−0.7425	2.31	0.1891		−1.27	2.03	0.2133		−0.2058	2.18	0.2001	
Residual												
Lack of Fit		3.98	0.2074	NS		2.73	0.2793	NS		2.10	0.3391	NS
R^2^			0.9357				0.9470				0.9235	
Adjusted R^2^			0.8199				0.8517				0.7859	

Statistical analyses were determined by one-way analysis of variance (ANOVA). *, *p* < 0.05; **, *p* < 0.01; NS: not statistically significant.

**Table 6:** The comparison of TPC, TFC, antioxidant, and isoflavone compounds (daidzein, genistein, and glycitein) between O-TFSE and TFSE.

Assay	O-TFSE	TFSE
**TPCs** (**mg of GAE/g DW**)	45.36 ± 0.62 ****	2.06 ± 0.02
**TFCs** (**mg of QE/g DW**)	3.16 ± 0.01 ****	0.11 ± 0.001
**Antioxidant**		
**ORAC** (**µmol of TE/g DW**)	2613.75 ± 26.60 ****	110.78 ± 1.43
**FRAP** (**µmol of TE/g DW**)	44.92 ± 2.19 ****	1.99 ± 0.10
**DPPH** (**µmol of TE/g DW**)	18.12 ± 1.21 ****	1.09 ± 0.04
**Isoflavone compounds**		
**Daidzein** (**mg/g extract**)	12.8 ± 0.03 ****	72.8 ± 0.04
**Genistein** (**mg/g extract**)	23.4 ± 0.19 ****	107.4 ± 0.24
**Glycitein** (**mg/g extract**)	3.2 ± 0.01 ****	6.8 ± 0.01

Results are shown as the mean ± standard deviation (SD) of triplicate experiments (n = 3). Statistical analysis was performed using an independent *t*-test to compare the optimized condition and the ethanol fraction condition. **** *p* < 0.0001 indicate statistically significant differences. GAE: gallic acid equivalent; QE: quercetin equivalent; TE: Trolox equivalent; DW: dry weight.

**Table 7:** Comparison of enzyme inhibition between O-TFSE, TFSE, and positive drugs (miglitol and acarbose).

Sample	% Inhibition	
	Alpha-Glucosidase	Alpha-Amylase
O-TFSE	72.91 ± 1.19 ***	64.31 ± 1.44 ****
TFSE	59.77 ± 1.37	37.09 ± 0.90
Miglitol	50.03 ± 0.20	ND
Acarbose	51.76 ± 1.60	48.34 ± 0.28

Results are shown as the mean ± standard deviation (SD) of triplicate experiments (n = 3). Statistical analysis was performed using an independent *t*-test to compare the optimized condition and the ethanol fraction condition. *** *p* < 0.001, **** *p* < 0.0001 indicate statistically significant differences. Alpha-glucosidase and Alpha-amylase using 6.25 mg/mL O-TFSE and TFSE concentrations. Alpha-glucosidase using 1.651 mg/mL miglitol and 0.895 mg/mL acarbose. Alpha-amylase using 11.053 µg/mL acarbose concentration. ND = Not determined.

**Table 8:** Receptor of *Saccharomyces cerevisiae* α-glucosidase (PDB: 3A4A) active site. (Constraints relaxed by 2.5 angstroms and 10 degrees).

Chemical Compounds	Vina Score (kcal/mol)	Grid Box (Å)	Size Box	Interactions with Enzyme
**Acarbose**	−8.94	22, −5, 23	40, 40, 40	TYP158 VAL216 ASP242
**Miglitol**	−5.80	22, −5, 23	40, 40, 40	ARG213 ASN350 ASP352 GLU411
**Daidzein**	−8.55	22, −5, 23	40, 40, 40	GLY161 ASN235 PHE432
**Genistein**	−8.36	22, −5, 23	40, 40, 40	SER241 SER311 ARG315
**Glycitein**	−8.41	22, −5, 23	40, 40, 40	HIS112 ARG213 ASP215 GLU277 HIS280

## Data Availability

The original contributions presented in the study are included in the article/Supplementary Materials. Further inquiries can be directed to the corresponding author.

## Supplementary Materials

The following supporting information can be downloaded at https://www.mdpi.com/article/10.3390/foods14152603/s1. Figure S1: HPLC chromatograms of (A) mixed standards of protocatechin (protocatechuic acid), daidzein, glycitein, and genistein; (B) TFSE and (C) O-TFSE; Figure S2: Fluorescent microscopic images of cells (A) displaying a bright field; cell morphology (left panel) and green fluorescence; and 2-NBDG signal (middle panel). The fluorescence signal is overlayed on the bright field image to visualize the co-localization of 2-NBDG within the cellular context (right panel). The cellular uptake of 2-NBDG (B). Relative fluorescence pixel intensity analyzed by Zeiss ZEN Pro software (version 3.9). Data are shown as the mean ± SD of three independent experiments. The differences among the treatment groups were determined using a one-way analysis of variance (ANOVA), followed by Tukey’s multiple comparison. * *p* Values < 0.05 vs. TNF-α-treated control; Table S1: Result of O-TFSE compound (positive mode cut off library score 95); Table S2: Result of O-TFSE compound (positive mode cut off library score 95).
