# Correction: Temviriyanukul et al. Enhancement of Phytochemicals and Antioxidant Activity of Thai Fermented Soybean Using Box–Behnken Design Guided Microwave-Assisted Extraction. *Foods* 2025, *14*, 2603

**DOI:** 10.3390/foods14183152

**Published:** 2025-09-10

**Authors:** Piya Temviriyanukul, Woorawee Inthachat, Ararat Jaiaree, Jirarat Karinchai, Pensiri Buacheen, Supachai Yodkeeree, Tanongsak Laowanitwattana, Teera Chewonarin, Uthaiwan Suttisansanee, Arisa Imsumran, Ariyaphong Wongnoppavich, Pornsiri Pitchakarn

**Affiliations:** 1 Institute of Nutrition, Mahidol University, Salaya 73170, Nakhon Pathom, Thailand; piya.tem@mahidol.ac.th (P.T.); woorawee.int@mahidol.ac.th (W.I.); uthaiwan.sut@mahidol.ac.th (U.S.); 2 Department of Biochemistry, Faculty of Medicine, Chiang Mai University, Muang Chiang Mai 50200, Chiang Mai, Thailand; ararat.ja@gmail.com (A.J.); jirarat.karin@gmail.com (J.K.); pensiri.bua@cmu.ac.th (P.B.); supachai.y@cmu.ac.th (S.Y.); tanongsak.l@cmu.ac.th (T.L.); teera.c@cmu.ac.th (T.C.); arisa.bonness@cmu.ac.th (A.I.); ariyaphong.w@cmu.ac.th (A.W.)

## Error in Figure

In the original publication [1], there was a mistake in Figure 1 as published. An incorrect image was used for Figure 1C during the proofreading process. The corrected Figure 1 appears below. The authors state that the scientific conclusions are unaffected. This correction was approved by the Academic Editor. The original publication has also been updated.

## Figures and Tables

**Figure 1:**
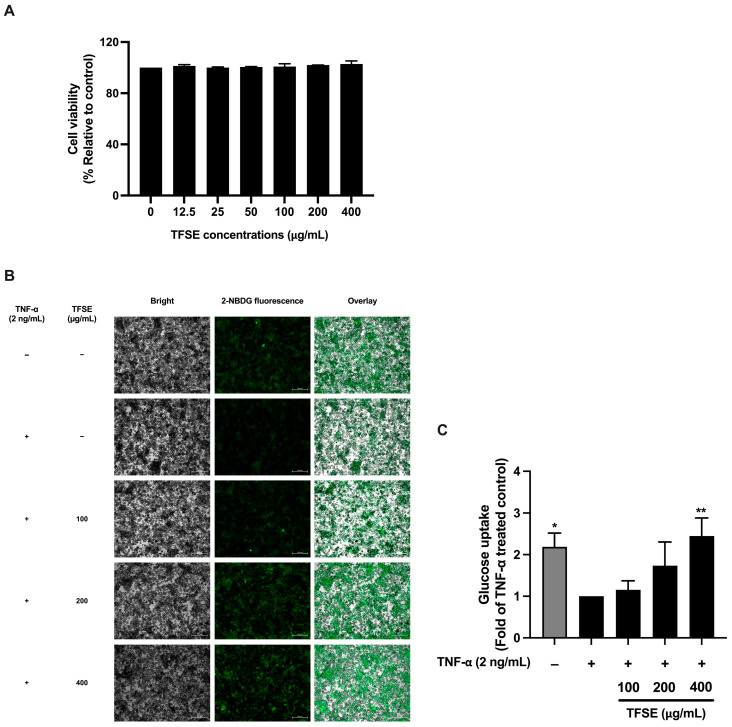
TFSE mitigates inflammation-induced insulin resistance. The cytotoxicity of TFSE in mature 3T3-L1 adipocytes determined by the SRB assay (**A**). The cells were treated with various doses of TFSE (12.5–400 µg/mL) for 72 h. The effect of TFSE on insulin-induced cellular glucose uptake in TNF-α-treated 3T3-L1 adipocytes (**B**,**C**). Fluorescent microscopic images of cells (**B**) displaying a bright field; cell morphology (left panel) and green fluorescence; and a 2-NBDG signal (middle panel). The fluorescence signal is overlayed on the bright field image to visualize the co-localization of 2-NBDG within the cellular context (right panel). The cellular uptake of 2-NBDG (**C**). Relative fluorescence pixel intensity analyzed by the Zeiss ZEN Pro software (version 3.9). The data are indicated as the mean ± SD of three independent experiments. The differences between the treatment groups were determined using a one-way analysis of variance (ANOVA), followed by Tukey’s multiple comparison. * *p* values < 0.05 and ** *p* values < 0.01 vs. TNF-α-treated control.
