# Supplementary material for: Enhancement of Phytochemicals and Antioxidant Activity of Thai Fermented Soybean Using Box–Behnken Design Guided Microwave-Assisted Extraction
Source: Foods. 2025 Jul 24;14(15):2603. doi: 10.3390/foods14152603 (PMC12346173; doi:10.3390/foods14152603)
Supplement: Supplementary file 1 [file foods-14-02603-s001.zip › foods-3750611-supplementary.pdf]

foods

Supplementary Materials

Enhancement of Phytochemicals and Antioxidant Activity of Thai Fermented Soybean Using Box–Behnken Design Guided Microwave-Assisted Extraction

Piya Temviriyanukul [1], Woorawee Inthachat [1], Ararat Jaiaree [2], Jirarat Karinchai [2], Pensiri Buacheen [2], Supachai Yodkeeree [2], Tanongsak Laowanitwattana [2], Teera Chewonarin [2], Uthaiwan Suttisansanee [1], Arisa Imsumran [2], Ariyaphong Wongnoppavich [2] and Pornsiri Pitchakarn [2,*]

[1] Institute of Nutrition, Mahidol University, Salaya, Nakhon Pathom 73170, Thailand; piya.tem@mahidol.ac.th (P.T.); woorawee.int@mahidol.ac.th (W.I.); uthaiwan.sut@mahidol.ac.th (U.S.)
[2] Department of Biochemistry, Faculty of Medicine, Chiang Mai University, Muang Chiang Mai, Chiang Mai 50200, Thailand; ararat.ja@gmail.com (A.J.); jirarat.karin@gmail.com (J.K.); pensiri.bua@cmu.ac.th (P.B.); supachai.y@cmu.ac.th (S.Y.); tanongsak.l@cmu.ac.th (T.L.); teera.c@cmu.ac.th (T.C.); arisa.bonness@cmu.ac.th (A.I.); ariyaphong.w@cmu.ac.th (A.W.)
[*] Correspondence: pornsiri.p@cmu.ac.th; Tel.: +66-53-935325

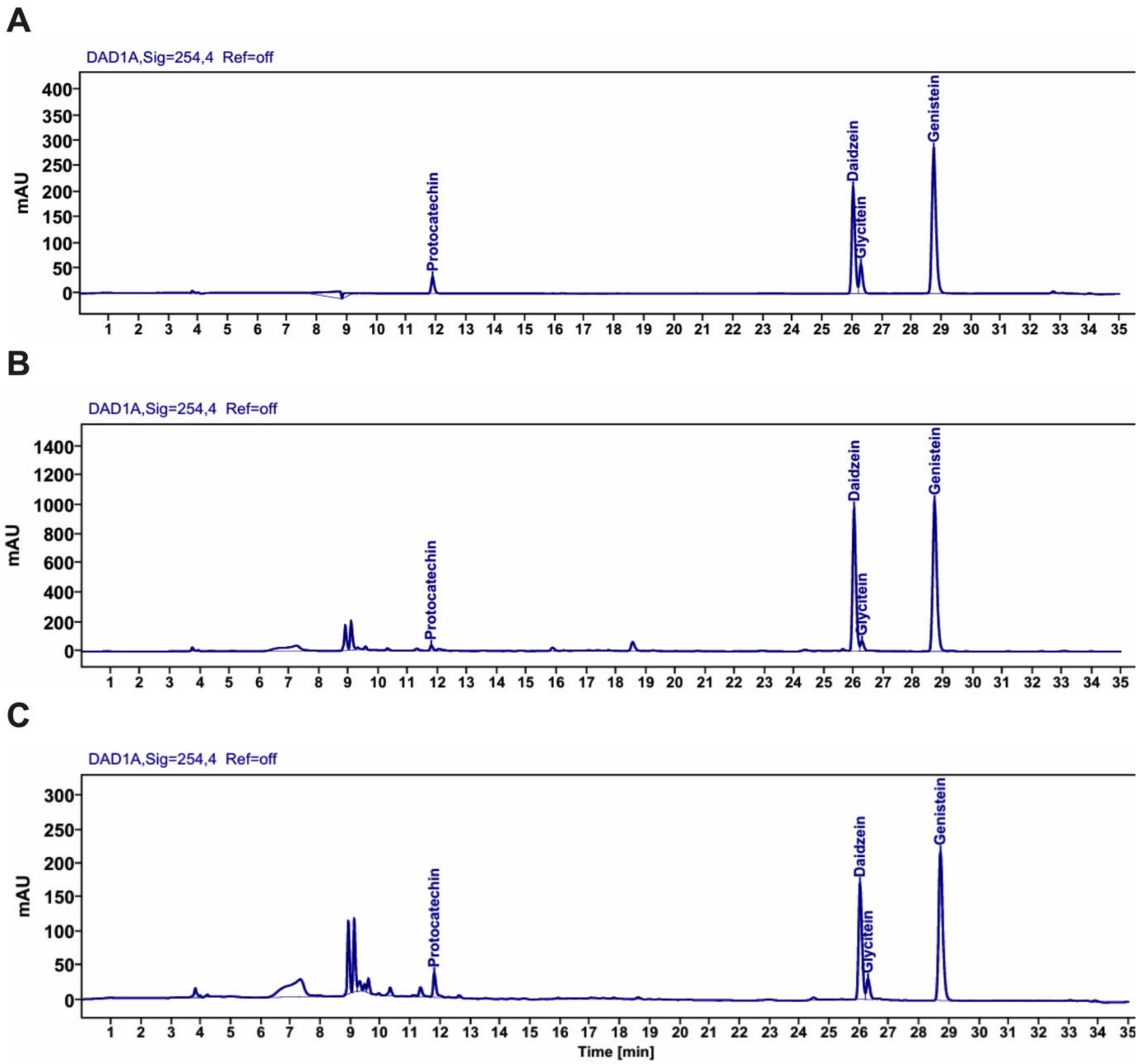

Figure S1. HPLC chromatograms of (**A**) mixed standards of protocatechin (protocatechuic acid), daidzein, glycitein, and genistein; (**B**) TFSE; and (**C**) O-TFSE.

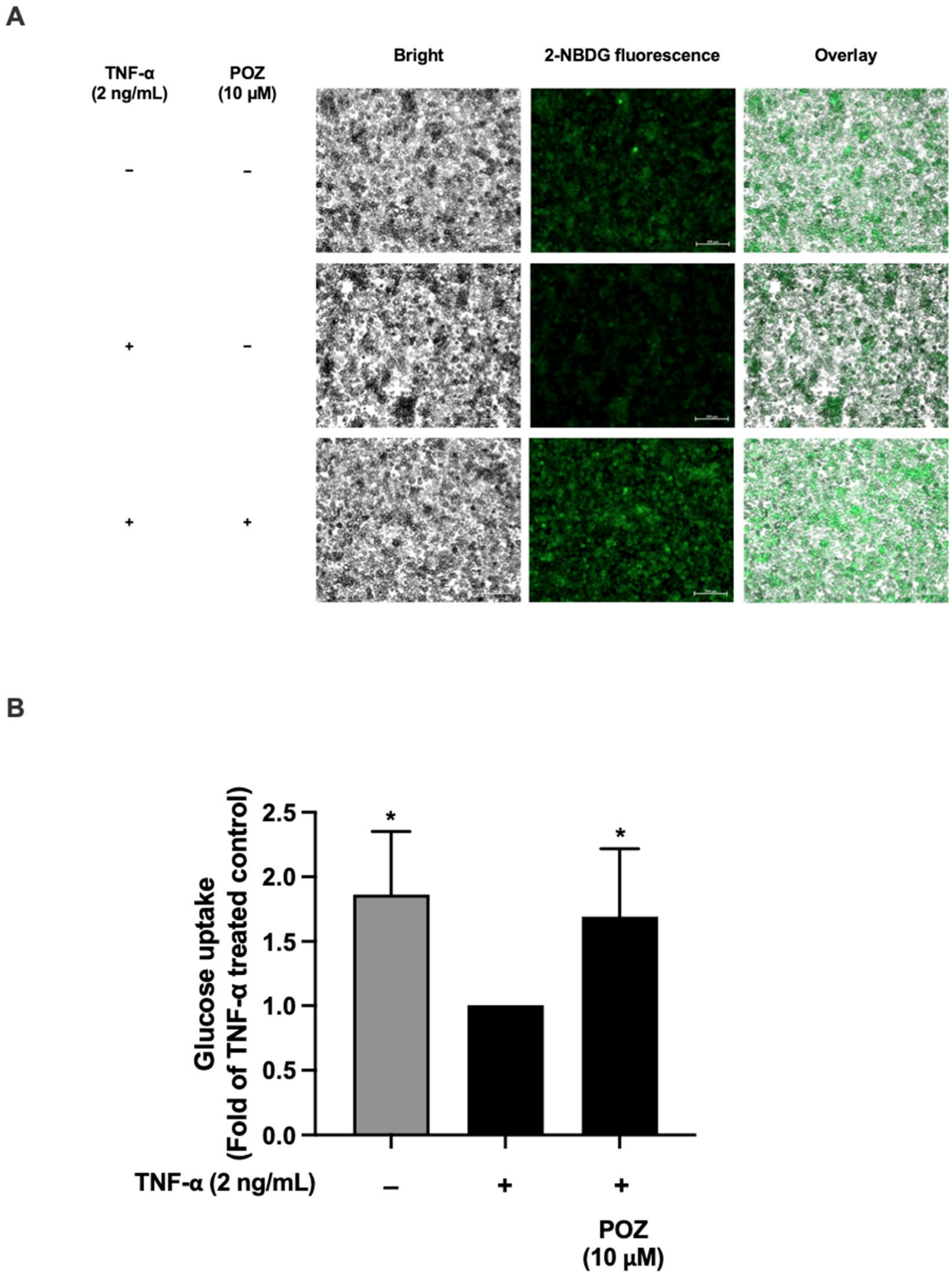

Figure S2. The effect of POZ (a positive control) on insulin-induced cellular glucose uptake in TNF-α-treated 3T3-L1 adipocytes. Fluorescent microscopic images of the cell (**A**), displaying bright field; cell morphology (left panel), and green fluorescence; 2-NBDG signal (middle panel). The fluorescence signal is overlayed on the bright field image to visualize co-localization of 2-NBDG within the cellular context (right panel). Cellular uptake of 2-NBDG (**B**). Relative fluorescence pixel intensity analyzed by the Zeiss ZEN Pro software (version 3.9). The data are indicated as mean ± SD of three independent experiments. The differences among the treatment groups were determined using a one-way analysis of variance (ANOVA), followed by Tukey's multiple comparison. * p values < 0.05 vs. TNF-**α**-treated control

Table S1. Result of compound of TFSE (Positive mode-cut off library score 95)

NO.	RT (min)	Tentative compounds	Adduct	Molecular mass	Library score	Relative area (%)
1	8.67	Daidzein	M+	254.0702	98.3	22.86
2	8.87	Glycetein	M+	284.07972	98.7	12.64
3	10.1	Genistein	M+	270.07056	97.5	11.25
4	2.27	Phenylalanine	M+	165.08823	100	10.60
5	1.51	Isoleucine	M+	131.10318	99.2	8.12
6	18.27	1-Palmitoyl-sn-glycero-3-phosphocholine	M+	495.34665	99.5	7.85
7	18.81	1-Oleoyl-sn-glycero-3-phosphocholine	M+	521.35675	98.9	5.25
8	25.6	Erucamide	M+	337.34643	95.3	5.19
9	4.63	2,3,5,6-Tetramethylpyrazine	M+H+	136.10703	98.5	2.18
10	1.04	L-Valine	M+	117.08562	95	2.06
11	20.09	1-Monolinoleoyl-rac-glycerol	M+H+	354.28415	96.2	1.60
12	3.53	Maltol	M+	126.03785	98.4	1.12
13	17.08	13-Keto-9Z,11E-octadecadienoic acid	M+H+	294.22764	95.7	1.11
14	20.96	Palmitamide	M+	255.26455	98.7	0.98
15	5.24	Ile-Leu	M+	244.18488	95.8	0.79
16	7	Genistin	M+K+	394.15586	99.5	0.61
17	9.95	Naringenin	M+	272.07329	97.7	0.56
18	1.2	Adenine	M+	135.06088	99.8	0.51
19	18.75	Monolinolenin	M+	352.27471	98.2	0.44
20	1.33	Hypoxanthine	M+	136.04426	100	0.41
21	23.19	Stearamide	M+	283.29436	98.2	0.34
22	1.02	Proline	M+	115.06794	98.5	0.32
23	1.42	Ile-Thr	M+	232.14702	97.1	0.32

24	1.01	Trigonelline	M+	137.05296	99.4	0.30
25	3.31	Phenylethylamine	M+	121.09452	99.1	0.26
26	6.06	Daidzin	M+	416.11591	99.2	0.26
27	0.87	Histidine	M+	155.07638	98.9	0.24
28	1.06	L(+)-Arginine	M+	174.1237	97.1	0.23
29	4.02	Phe-Ala	M+CH3OH+H+	204.09596	96.3	0.20
30	4.09	Phenprobamate	M+	165.0838	98.7	0.17
31	1.01	Pipecolinic acid	M+	129.09119	95.4	0.17
32	4.01	1H-Indole-4-carboxaldehyde	M+	145.05918	97.7	0.16
33	1.23	Guanine	M+	151.10611	95.7	0.15
34	3.92	Phe-Gly	M+NH4+	205.08026	98.4	0.14
35	23.36	1-Stearoyl-rac-glycerol	M+	358.31417	95.9	0.13
36	16.79	Benzyldodecyldimethylammonium cation	M+	303.29897	95.3	0.12
37	15.81	1-Stearoyl-2-hydroxy-sn-glycero-3-phosphocholine	M+	523.36922	99.3	0.09
38	10.2	Tectorigenin	M+	300.07498	99.4	0.08
39	11.2	Formononetin	M+	268.07945	99.2	0.07
40	1.08	Glutamic acid	M+	147.10595	100	0.06
41	12.93	Biochanin A	M+	284.07605	99.2	0.04
42	21.22	1-Palmitoyl-2-azelaoylphosphatidylcholine	M+	665.4297	98.3	0.01
43	12.24	9-Deoxy-9-methylene-16,16-dimethylprostaglandin E2	M+	360.21232	95.9	0.01

Table S2. Result of compound of O-TFSE (Positive mode-cut off library score 95).

NO.	RT (min)	Tentative compounds	Adduct	Molecu-lar mass	Library score	Relative area (%)
**1**	1.85	Phenylalanine	M+	165.12	100	16.45
**2**	10.11	Genistein	M+	270.06	97.5	15.08
**3**	8.42	Daidzein	M+	254.07	97.4	13.63
**4**	19.06	1-Monolinoleoyl-rac-glycerol	M+	354.29	96.2	10.95
**5**	1.48	Isoleucine	M+	131.1	99.1	7.41
**6**	9	Glycetein	M+	284.08	97.6	6.33
**7**	17.94	Monolinolenin	M+	352.27	97.9	4.39
**8**	12.05	Soyasaponin	M+	942.54	96.8	4.06
**9**	20.13	Monoolein	M+	356.3	95.9	3.47
**10**	14.9	Bergaptol	M+	202.04	99.2	1.50
**11**	19.46	Palmitamide	M+	255.26	98.5	1.46
**12**	4.69	Val-Leu	M+	230.17	95.3	1.15
**13**	1	Arginine	M+	174.12	97.1	0.91
**14**	4.07	1H-Indole-4-carboxaldehyde	M+	145.06	98.1	0.85
**15**	1.02	Stachydrine	M+	143.11	100	0.81
**16**	3.74	Val-Ile	M+	230.17	98.2	0.71
**17**	20.04	2-Oleoyl-1-palmitoyl-sn-glycero-3-phosphocholine	M+	759.59	100	0.70
**18**	3.97	Phe-Gly	M+	222.1	98.3	0.63
**19**	3.18	Phenylethylamine	M+	121.09	99.3	0.58
**20**	0.99	Proline	[M+H]+	115.07	98.5	0.56
**21**	2.84	Leu-Gly	M+	188.12	100	0.53
**22**	26.78	1,2-dioleoyl-sn-glycero-3-phosphatidylcholine	M+	785.61	99.6	0.50

**23**	5.58	Tyr-Leu	M+	294.16	96.8	0.48
**24**	1.39	Tyramine	M+	137.09	95.2	0.44
**25**	1.22	Guanine	[M+H]+	151.05	95.2	0.43
**26**	14.9	Isoimperatorin	M+	270.1	98.6	0.41
**27**	9.12	Naringenin	M+	272.07	97.7	0.37
**28**	1.34	6-Hydroxypurine	[M]+	137.05	100	0.37
**29**	0.86	Pipecolinic acid	[M+H]+	129.08	98.1	0.37
**30**	4.21	Phe-Ala	[M+CH3OH+H]+	204.09	95.8	0.36
**31**	0.87	Histidine	M+	155.07	98.4	0.36
**32**	17.68	7-Hydroxycoumarin	M+	162.04	97.6	0.36
**33**	1.8	Phe-Ser	M+	252.12	95.5	0.31
**34**	3.51	Maltol	M+	126.04	97.9	0.29
**35**	15.45	N-Docosanoyl-4-sphingenyl-1-O-phosphorylcholine	M+	786.61	98.4	0.28
**36**	23.33	Erucamide	M+	337.34	96.2	0.27
**37**	16.79	Phthalic anhydride	M+	148.02	95.7	0.25
**38**	0.98	Glutamic acid	[M+CH3OH+H]+	115.03	100	0.25
**39**	0.86	L-Lysine	[M+NH4]+	129.08	95.1	0.24
**40**	0.99	Trigonelline	[M+H]+	137.05	99.4	0.22
**41**	10.91	Piperitone	M+	152.12	95.5	0.15
**42**	4.71	Phe-Val	M+	264.1	96.3	0.15
**43**	1.94	Phe-Thr	M+	266.13	98.1	0.14
**44**	12.07	Oxypeucedanin	M+	286.09	98	0.13
**45**	10.25	Tectorigenin	M+	300.07	95.6	0.09
**46**	7.81	Rhoifolin	M+	578.17	99.3	0.08
**47**	21.32	Stearamide	[M+NH4]+	266.27	97.4	0.08

**48**	1.01	D-Ornithine	[M+NH4]+	115.03	96.6	0.07
**49**	10.91	Xanthotoxol	M+	202.03	99.2	0.07
**50**	1.31	Uracil	M+	112.03	98.9	0.06
**51**	26.78	Arachidonoylthiophosphorylcholine	M+	783.59	99.6	0.05
**52**	1.39	Adenosine	M+	267.11	97.3	0.04
**53**	11.24	Formononetin	M+	268.09	97.5	0.04
**54**	26.78	1-Palmitoyl-2-linoleoyl-sn-glycero-3-phosphocholine	M+	575.57	99.3	0.03
**55**	1.42	guanosine	M+	283.1	99.4	0.03
**56**	15.32	.alpha.-Cyperone	M+	218.17	95.4	0.02
**57**	9.24	Oxypeucedanin hydrate	M+	304.1	97.5	0.01
**58**	7.85	Neodiosmin	M+	608.18	96.3	0.01
